# COVID-19 machine learning model predicts outcomes in older patients from various European countries, between pandemic waves, and in a cohort of Asian, African, and American patients

**DOI:** 10.1371/journal.pdig.0000136

**Published:** 2022-11-08

**Authors:** Behrooz Mamandipoor, Raphael Romano Bruno, Bernhard Wernly, Georg Wolff, Jesper Fjølner, Antonio Artigas, Bernardo Bollen Pinto, Joerg C. Schefold, Malte Kelm, Michael Beil, Sviri Sigal, Susannah Leaver, Dylan W. De Lange, Bertrand Guidet, Hans Flaatten, Wojciech Szczeklik, Christian Jung, Venet Osmani

**Affiliations:** 1 Digital Health Centre, Fondazione Bruno Kessler Research Institute, Trento, Italy; 2 Heinrich-Heine-University Duesseldorf, Medical Faculty, Department of Cardiology, Pulmonology and Vascular Medicine, Duesseldorf, Germany; 3 Department of Internal Medicine, General Hospital Oberndorf, Teaching Hospital of the Paracelsus Medical University Salzburg, 5020 Salzburg, Austria; 4 Institute of General Practice, Family Medicine and Preventive Medicine, Paracelsus Medical University, Salzburg, Austria; 5 Department of Anaesthesia and Intensive Care, Viborg Regional Hospital, Viborg, Denmark; 6 Department of Intensive Care Medicine, CIBER Enfermedades Respiratorias, Corporacion Sanitaria Universitaria Parc Tauli, Autonomous University of Barcelona, Sabadell, Spain; 7 Department of Acute Medicine, Geneva University Hospitals, Geneva, Switzerland; 8 Department of Intensive Care Medicine, Inselspital, Universitätsspital, University of Bern, Bern, Switzerland; 9 Dept. of Medical Intensive Care, Hadassah Medical Center and Faculty of Medicine, Hebrew University of Jerusalem, Israel; 10 General Intensive care, St George’s University Hospitals NHS Foundation trust, London, United Kingdom; 11 Department of Intensive Care Medicine, University Medical Center, University Utrecht, the Netherlands; 12 Sorbonne Universités, UPMC Univ Paris 06, INSERM, UMR_S 1136, Institut Pierre Louis d’Epidémiologie et de Santé Publique, Equipe: épidémiologie hospitalière qualité et organisation des soins, F-75012, Paris, France; 13 Assistance Publique—Hôpitaux de Paris, Hôpital Saint-Antoine, service de réanimation médicale, Paris, France; 14 Department of Clinical Medicine, University of Bergen, Department of Anaesthesia and Intensive Care, Haukeland University Hospital, Bergen, Norway; 15 Jagiellonian University Medical College, Center for Intensive Care and Perioperative Medicine, Krakow, Poland; University of Cagliari: Universita degli Studi Di Cagliari, ITALY

## Abstract

**Background:**

COVID-19 remains a complex disease in terms of its trajectory and the diversity of outcomes rendering disease management and clinical resource allocation challenging. Varying symptomatology in older patients as well as limitation of clinical scoring systems have created the need for more objective and consistent methods to aid clinical decision making. In this regard, machine learning methods have been shown to enhance prognostication, while improving consistency. However, current machine learning approaches have been limited by lack of generalisation to diverse patient populations, between patients admitted at different waves and small sample sizes.

**Objectives:**

We sought to investigate whether machine learning models, derived on routinely collected clinical data, can generalise well i) between European countries, ii) between European patients admitted at different COVID-19 waves, and iii) between geographically diverse patients, namely whether a model derived on the European patient cohort can be used to predict outcomes of patients admitted to Asian, African and American ICUs.

**Methods:**

We compare Logistic Regression, Feed Forward Neural Network and XGBoost algorithms to analyse data from 3,933 older patients with a confirmed COVID-19 diagnosis in predicting three outcomes, namely: ICU mortality, 30-day mortality and patients at low risk of deterioration. The patients were admitted to ICUs located in 37 countries, between January 11, 2020, and April 27, 2021.

**Results:**

The XGBoost model derived on the European cohort and externally validated in cohorts of Asian, African, and American patients, achieved AUC of 0.89 (95% CI 0.89–0.89) in predicting ICU mortality, AUC of 0.86 (95% CI 0.86–0.86) for 30-day mortality prediction and AUC of 0.86 (95% CI 0.86–0.86) in predicting low-risk patients. Similar AUC performance was achieved also when predicting outcomes between European countries and between pandemic waves, while the models showed high calibration quality. Furthermore, saliency analysis showed that FiO2 values of up to 40% do not appear to increase the predicted risk of ICU and 30-day mortality, while PaO2 values of 75 mmHg or lower are associated with a sharp increase in the predicted risk of ICU and 30-day mortality. Lastly, increase in SOFA scores also increase the predicted risk, but only up to a value of 8. Beyond these scores the predicted risk remains consistently high.

**Conclusion:**

The models captured both the dynamic course of the disease as well as similarities and differences between the diverse patient cohorts, enabling prediction of disease severity, identification of low-risk patients and potentially supporting effective planning of essential clinical resources.

**Trial registration number:**

NCT04321265.

## Author summary

COVID-19 remains a complex disease, making it challenging to estimate the risk of deterioration of critically ill patients and consequently allocation of clinical resources, such as ventilators. As a result, there is a need to support clinical decision making through objective methods and address some of the limitations of the current clinical scoring systems. In response, we developed machine learning models using routine clinical data of patients from 37 countries worldwide, including 18 European countries. We find that: i) machine learning models can predict outcomes in patients from diverse European countries, that is which patients have a low risk of deterioration and which may require increased care such that resources are allocated efficiently; ii) routine clinical data from European patients can be used to predict outcomes in non-European patients, namely those admitted in Asian, African, and American intensive care units, without significantly affecting the performance, and iii) routine clinical data collected during the first COVID-19 pandemic wave, can be used to predict the risk of deterioration of patients admitted during subsequent waves. Our study is the first step towards improving standardisation and equity of critical care across healthcare institutions and further afield across diverse countries and territories.

## Introduction

The coronavirus pandemic continues to strain health care systems globally [1]. While much has been discovered about the disease aetiology, many open questions remain around disease trajectories, considering diverse patient outcomes in terms of mortality rate as well as the need for ventilation. Several studies [2–4] have found that 3% to 79% of hospitalised patients required invasive mechanical ventilation (MV), with a significant heterogeneity in ICU outcomes [5]. These aspects render clinical resource allocation challenging to plan. Early risk stratification can help in early identification of patients with a high risk of deterioration and adjust treatment course. However, varying symptomatology, especially pronounced in older patients, still includes several unknowns. Furthermore, the current scoring systems in clinical practice are limited by small sample size and consequently have low predictive power, especially for prediction of mortality in COVID-19 patients [6].

More objective and consistent methods are required that can assist clinicians in discriminating between patients with low risk of deterioration and those that may require increased care, estimating risk in a continuous manner considering evolution of the patients’ state as well as administration of therapeutical interventions. Assisting clinicians in this manner becomes crucial for countries with limited resources and varying expertise, especially facing a novel disease (such as COVID-19) where reliable models to guide effective allocation of essential resources and improve patient outcomes are scarce [7]. Furthermore, more objective assessment methods have the potential to mitigate inequalities in allocation of medical resources [8,9].

Machine learning approaches have shown the potential to enhance prognostication, by capturing non-linear relationships between variables to predict outcomes of interest. However, current efforts have been limited by lack of generalisation to diverse patient populations, between patients admitted at different waves and small sample sizes. While there are many studies investigating prediction of outcomes in COVID-19 patients [10–14], only a handful have investigated generalisability of the models across countries with diverse populations located in different continents using imaging [15] and no studies have been found that used routinely collected data, as outlined in [15]. Indeed, a recent review on chest imaging, emphasised the importance of validation dataset to assess generalisability of the model to other cohorts, rather than only on the sampled population [16].

Therefore, the main objective of this work is to investigate whether the use of routinely collected Electronic Health Records (EHR) data in older patients with COVID-19, coupled with machine learning (ML) algorithms can generalise to diverse patients’ populations, to estimate the risk of ICU and 30-day mortality, as well as identify patients at low risk of deterioration, likely to survive without a therapeutic intervention. We hypothesised that a machine learning model derived in a cohort of COVID-19 older patients can be used to predict clinically relevant outcomes of both, geographically and temporally (between pandemic waves) diverse cohorts.

## Methods

To address our hypothesis, we developed and validated several machine learning models, derived from data collected from 3,933 older patients with a confirmed COVID-19 diagnosis, admitted to ICUs located in 37 countries, between January 11, 2020, and April 27, 2021 as part of the COVIP study (trial registration number NCT04321265, March 25, 2020). We evaluate the resulting models in a i) *retrospective study* with validation between the European countries to assess inter-country generalisability of the European model; ii) *prospective study* between the pandemic waves to evaluate the ability of the model derived from a cohort of patients admitted during a COVID-19 wave to generalise in predicting outcomes in patients admitted to European ICUs during the subsequent wave; and iii) *external validation* in a cohort of non-European patients, to evaluate whether the model derived from the overall European cohort can be used to predict outcomes in highly diverse patients, such as those admitted to Asian, African and American ICUs.

For each of the three study designs, we investigated whether the models’ predicted probabilities match the actual observed probabilities of each of the three outcomes, namely quality of the model calibration. We also performed saliency analysis to identify the top-ranked variables that contributed most to the prediction of each of the three outcomes of interest for each study design.

### Outcomes definition

Primary outcomes in this study were: 1) mortality prediction, either in the ICU or 30 days after ICU admission; and 2) early identification of patients at low risk of deterioration, defined as patients who survived in the ICU without receiving any therapeutic intervention (i.e. invasive or non-invasive mechanical ventilation, administration of vasopressors, renal replacement therapy, and tracheostomy).

### Study design

**Fig 1:**
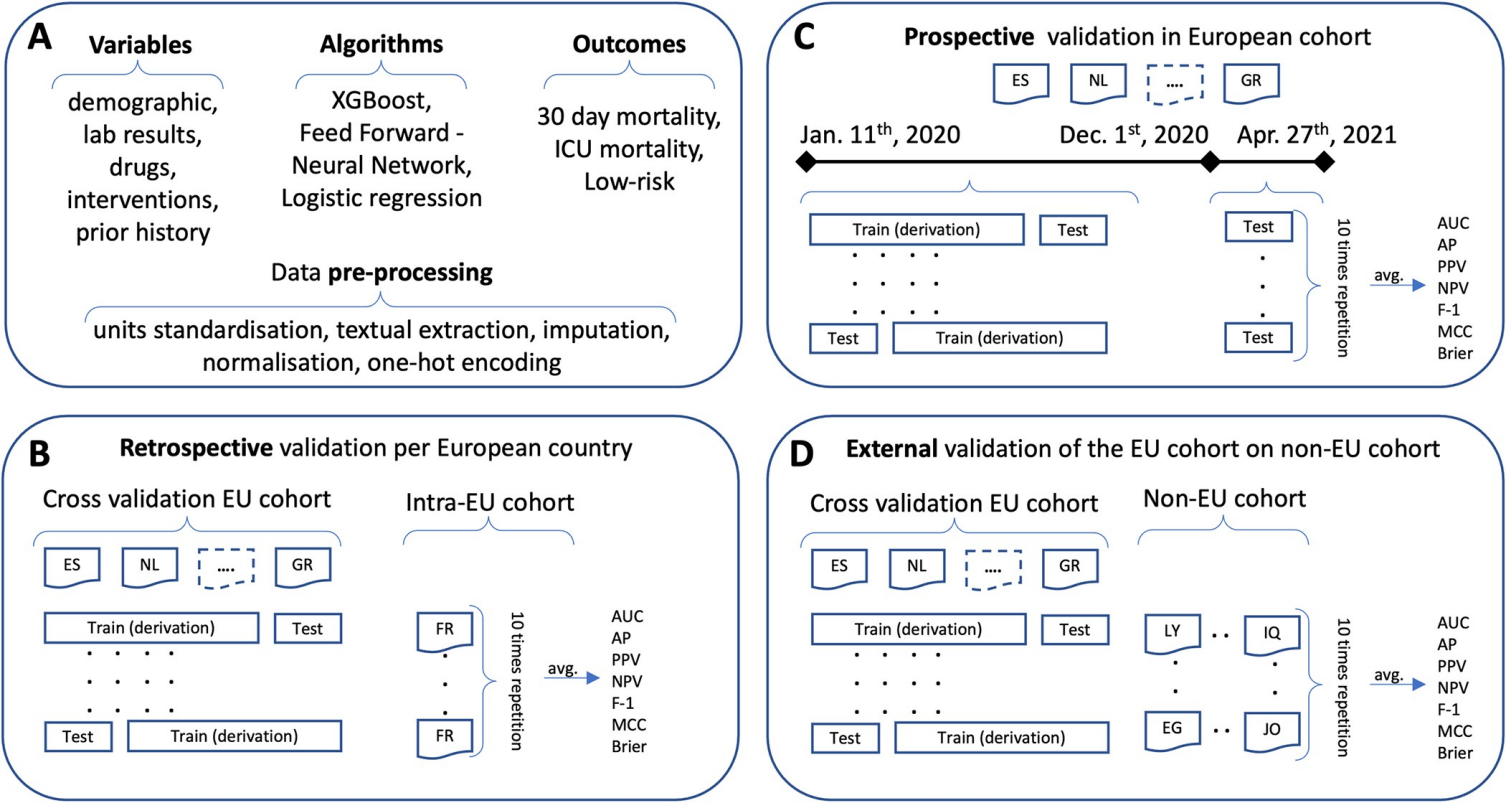
Description of variables, algorithms, and outcomes as well as pre-processing steps for the overall dataset (panel A). Retrospective validation of the model derived on the European cohort based on cross-validation as well as external validation on France as the country with highest ICU admissions (panel B), while the results for the rest of the European countries are shown in Tables A-C in S6 Text. Prospective validation of the model derived on the European cohort of patients admitted to ICUs before the cut-off date (December 1st, 2020) and validated on the European cohort of patients admitted to ICUs after the cut-off date, during the subsequent pandemic wave (panel C). External validation of the model derived on the overall European cohort and validated on the cohort of patients admitted to Asian, African and American ICUs (panel D). Note, EU is an abbreviation of Europe.

Our analysis primarily focuses on the European patient cohort, while we used the non-European cohort as the external validation dataset to investigate the generalisability of the models when encountering diverse patient populations, such as those from different continents. The overall workflow and study design is depicted in Fig 1.

**Fig 2:**
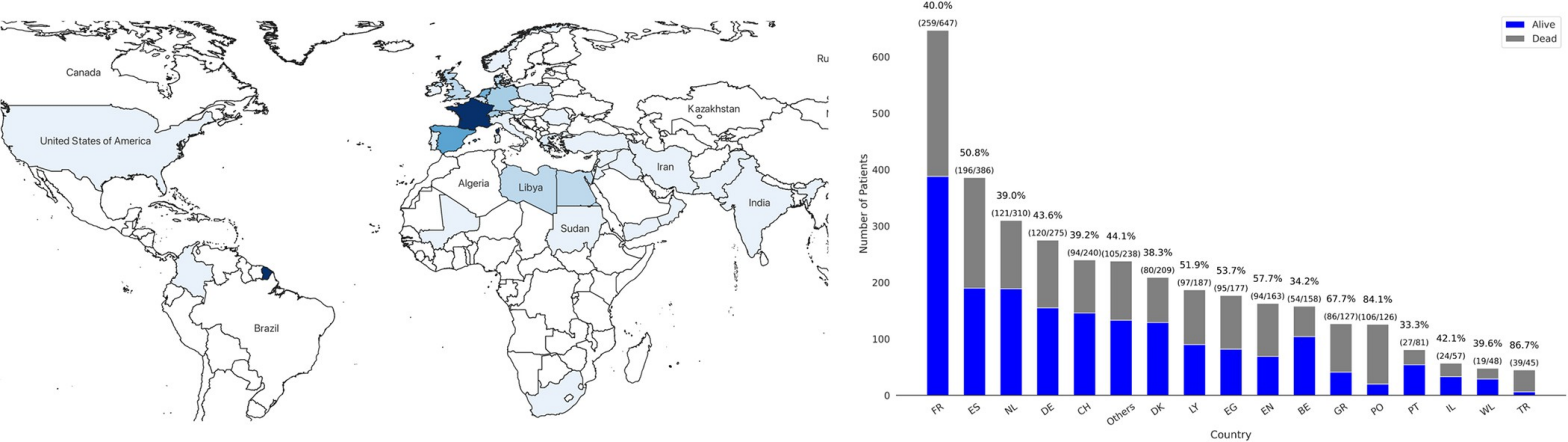
Map of the countries and continents represented in our dataset (left panel). Number of patients for the overall cohort as well as mortality rate per country (right panel). Country and territory abbreviations are detailed in S9 Text. (Map created with QGIS v.3.26 based on data from Natural Earth).

Initially, we retrospectively assessed the generalisability of the models among the European cohort, evaluating the predictive performance of the models derived from 16 European countries (as shown in Fig 2) using the patient cohort from France (that had the highest number of ICU admissions) as the validation. Furthermore, we evaluated inter-country generalisability of the European model, by evaluating its performance on top-nine European countries (based on the highest number of ICU admissions) separately, each time deriving the model from the patient cohorts of the remaining countries, in a leave one country out approach.

Following from this, we prospectively assessed the temporal generalisability of the models between different waves, by deriving a model from a patient cohort admitted before December 1^st^, 2020 (our cut-off date) and validating in a cohort of patients admitted on or after December 1^st^, 2020. The cut-off date was chosen based on the availability of the data as well as peak of cases per million in Europe between the first wave (peaking on November 7^th^, 2020) and the subsequent wave (peaking around January 11^th^, 2020) as shown in Fig C in S2 Text.

Lastly, we externally evaluated the predictive performance of the models derived from the overall European patient cohort to assess their generalisability in a validation cohort of patients admitted to Asian, African, and American ICUs.

For each study design we defined three outcomes of interest, namely ICU mortality, 30-day mortality and identification of low-risk patients. For the internal evaluation of each study design, namely of the European cohort leaving out France, European cohort admitted before the cut-off date December 1st, 2020, and the overall European cohort, we used stratified 5-fold cross-validation with 10 times repetition, starting with different initial random states to mitigate the randomness effects of a single train-test split.

### Ethics approval and consent to participate

The study was approved by the Ethics Committee of the University of Duesseldorf, Germany. Institutional research ethic board approval was obtained from each study site, as a prerequisite for participation in the study.

### Clinical data sources and settings

The study included older patients (over 70 years) admitted to ICUs originating from 37 countries around the world, with a confirmed diagnosis of COVID-19 based on a positive polymerase chain reaction (PCR) test. National coordinators of the study oversaw ICUs recruitment, obtaining national and local ethical approval, and supervising patient recruitment. Ethical approval was mandatory to participate in the study. The study was in line with the European Union General Data Privacy Regulation (GDPR) directive as part of the multi-centre COVIP clinical trial (ID: NCT04321265), where a database was established to facilitate the information sharing of electronic case report forms (eCRF) of each subject on a secure server at the Aarhus University, Denmark.

### Study population

All the patients involved in this study were at least 70 years old, admitted to 217 different ICUs from 172 cities in 37 independent countries between January 11, 2020, and April 27, 2021. The dataset included overall 3,933 patients with a unique eCRF record since each patient could only be entered into the database once regardless of their transfer to another ICU or readmission. After applying the selection criteria 3,474 patients remained, out of which 2,858 patients were admitted in European ICUs, while 616 patients were admitted in Asian, African and American ICUs. Patients were excluded due to having negative sarscov2 test and significant missing information, as shown in selection criteria diagram in S8 Text. All patients were followed up through a phone interview for their survival status after 30-days and 3-months from the ICU discharge.

### Study data and variables of interest

All the participating centres reported the patients’ information using consistent electronic case report forms (eCRF). Collected demographic information included age, sex, height, weight, and BMI. Furthermore, information about the presence of symptoms before hospitalization and the duration of hospital stays before ICU admission were also recorded.

Sub-scores of sequential organ failure assessment (SOFA): respiratory, cardiovascular, hepatic, coagulation, renal, and neurological systems were calculated at the ICU admission. Six different pre-existing comorbidities were also documented in eCRF form: diabetes, ischemic heart disease, renal insufficiency, arterial hypertension, pulmonary comorbidity, and congestive heart failure. The definitions of these comorbidities are available in S1 Text.

Several laboratory measurements were also retrieved for the patients during their ICU admission. The partial pressure of oxygen (PaO2) and the fraction of inspired oxygen (FiO2) were recorded based on the first arterial blood gas (ABG) analysis. The highest measured values of serum bilirubin, serum creatinine, c-reactive protein, and leukocytes count were documented on admission day. The serum lactate concentration was reported on both the first and second days of ICU admission separately. Furthermore, the minimum available measurements of thrombocyte count and lymphocyte count were also recorded.

Information on drug therapy during patients’ ICU stays included antibiotics, corticosteroids, and antiviral drugs, while also documenting bacterial co-infection. Finally, therapeutic interventions including invasive and non-invasive ventilation, vasopressor use, renal replacement therapy, tracheostomy, as well as their day of occurrence after ICU admission were also available in the dataset.

### Statistical analysis

We analysed baseline characteristics of patients using medians (IQRs) for continuous variables and frequencies (percentages) for categorical variables. We used the Kruskal–Wallis test (ANOVA) for continuous variables and the chi-square test for categorical variables to compare subgroups of alive and deceased patients.

### Data preparation

After removing inconsistently recorded information, we standardised variables into unique measurement units. For example, we converted PaO2 values with kilopascals (kPa) to millimetres of mercury (mmHg). Then we transformed ICU length of stay, mortality time and intervention start time into daily units. We converted textual notes of drug usage into binary variables indicating whether a drug was administered. We also extracted the SOFA sub-scores for each patient from their textual clinical notes and converted them to 6 separate variables with values ranging from 0 to 4. However, in the case of an incorrectly reported text, they were considered as missing values. We also checked the total SOFA score for consistency, summing all the sub-scores.

To improve interpretability, we defined four clinically meaningful intervals for each intervention, namely 0 to 2 days, 3 to 6 days, 7 to 30 days, and after 30 days, which then became separate binary variables. Furthermore, to mitigate the effect of noisy data and outliers in the dataset, we defined clinically valid intervals for the relevant variables and those variables outside of the intervals were considered as missing values. Since machine learning models typically cannot handle missing data, we imputed the missing information of patients in the dataset in several steps. We considered unreported values of bacterial co-infections and those reported as “unknown” as missing values. Then, we used the median for continuous and mode for categorical variables to impute the missing values in the rest of the dataset. Finally, we transformed each continuous variable individually within a zero to one range, maintaining their distribution, while we encoded the rest of the categorical variables using the one-hot encoding scheme.

To mitigate potential data leakage during the model derivation and validation, all the pre-processing steps were conducted after the data was split in train-test sets. Consequently, during the internal 5-fold cross-validation experiment design, the patients were divided into training folds and test folds first, and only then all the transformation steps were applied over the data. Furthermore, we excluded variables indicating clinical therapeutic interventions after 30 days for the 30-day mortality prediction outcome; and excluded all the variables that indicated therapeutic intervention when predicting the low-risk outcome. We also used the 3-month outcome to ensure consistency of the primary outcome and allay the concerns of censoring bias.

### Model development and validation

During model development we compared the performance of three algorithms, namely, Extreme Gradient Boosting (XGBoost) [17] as the primary model with Feed-Forward (FF) neural network and Logistic Regression (LR) [18] to predict ICU mortality, 30-days mortality after ICU admission, and low-risk patients admitted to the ICU. XGBoost is an ensemble of decision trees that provides robust predictive performance with learning complex and non-linear relationships in data using an ensemble learning technique called boosting. Boosting is an iterative learning process, sequentially building many models that correct the deficiencies of the preceding model. Even though deep neural networks provide better predictive performance in unstructured datasets, XGBoost has shown great predictive performance for structured, tabular data [19].

To compare the performance of XGBoost, we also implemented Feed-Forward as a deep neural network and Logistic Regression as a statistical baseline competitor. Feed-Forward model was a two-layer neural network with 64 and 16 neurons in the first and second layer respectively, using sigmoid activation function. Model parameters were randomly initialized based on Xavier normal method, trained for 100 epochs with batch size 32, and optimized using the Adam optimizer algorithm. Logistic Regression is a statistical method, investigates the relation of the outcome variable with the input variables, and typically considered as a baseline algorithm in clinical classification tasks.

All the three models were tuned for the best hyperparameters on the internal evaluation cohorts in each study design and outcome definitions. The models’ hyperparameters were optimized through exhaustive grid-search for maximizing the F-1 score metric and set for the final internal and external evaluation.

### Experimental evaluation

Training and evaluation of the models was based on 5-fold stratified cross-validation with 10-times repetition starting with different random states. Stratification ensures that outcome distribution in each fold is representative of the distribution of outcomes across the entire study population. Predictive performance of the models was evaluated using area under the receiver operator characteristic curve (AUC) and area under the precision-recall curve (AUPRC). Furthermore, since machine learning models can be discriminative but with low calibration quality, the calibration curve was plotted for all the analyses. The calibration curve shows the actual class probabilities against the models’ probability predictions and is evaluated using Brier scores (a lower Brier score indicates higher calibration quality). To assess the predictive performance, additional metrics were also calculated, including Positive Predictive Value (PPV), Negative Predictive Value (NPV), F-1 score, and Matthews correlation coefficient (MCC), shown in S4 Text. We note that in addition to MCC that considers the class imbalance [20], other methods could also be applicable, such as partial AUC [21] or subgroup analysis [22].

### Model interpretation

We used SHAP (Shapley Additive exPlanations) to interpret the output of the predictive models [23]. SHAP is a powerful method that explains how the model makes individual predictions by deconstructing every prediction into the sum of contributions from each input variable, known as SHAP values. SHAP values are a game-theoretic approach to model interpretability revealing how the input variables influence the final model’s predictions at the instance level and throughout the entire population.

In this study a SHAP value was calculated for each run of the 5-fold cross-validation (repeated 10 times) to precisely capture the influence of each variable during the model evaluation. These values were then plotted into a Bee swarm plot, an informative display of SHAP values that shows the relative importance of variables and their actual relationships with the predicted outcome.

## Results

### Study population

The overall dataset contained 3,933 electronic health records of patients, out of which 3,474 patients remained after applying the exclusion criteria as shown in the cohort selection diagram in S8 Text. The final cohort contained patients originating from 37 different countries (17 European and 20 non-European), admitted to ICUs between January 11, 2020, and April 27, 2021, shown in Fig 2.

**Fig 3:**
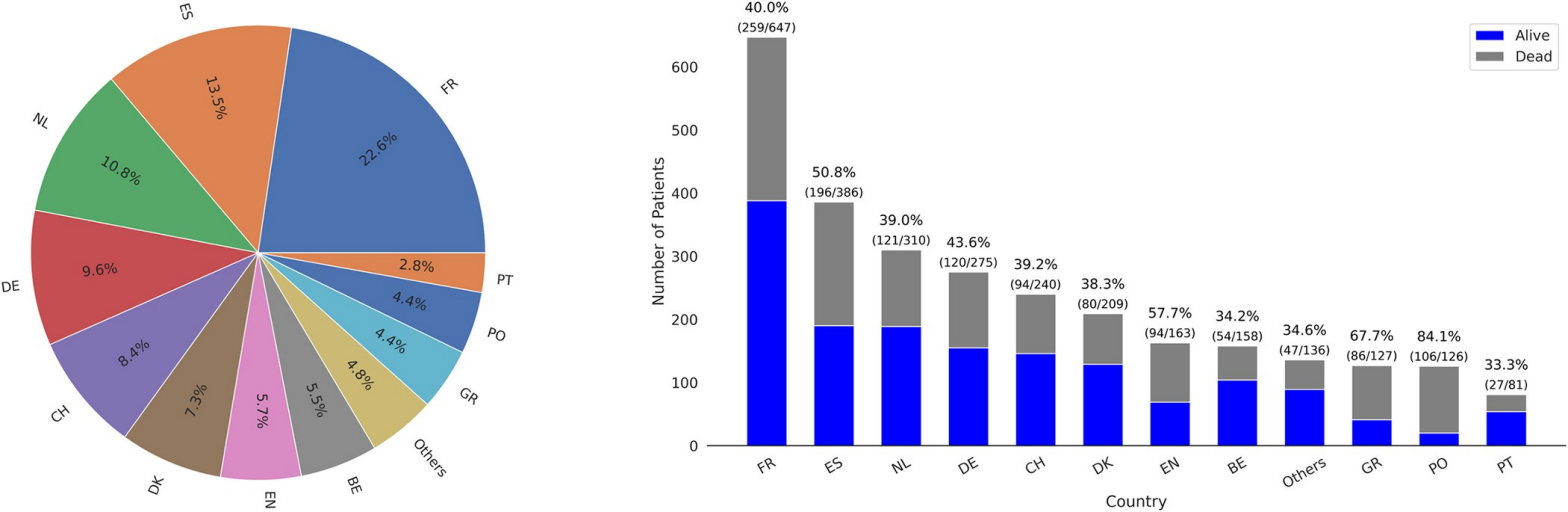
Distribution of patients admitted to European ICUs as a percentage (%) of the overall European cohort (left panel). Number of patients admitted to European ICUs as well as the mortality rate per European country, shown as a ratio between survivors (blue) and non-survivors (grey) (right panel). Country and territory abbreviations are detailed in S9 Text.

The European cohort included 2,858 patients with an average mortality rate of 45% both in ICU and 30 days after ICU admission, while 13% of patients were at low risk of deterioration. European patients’ age median was 75 years (IQR, [72–78]), with 30% female, and median length of ICU stay was 13 days (IQR, [6–22]). The distribution of patients among the European countries, including the number of patients as well as ICU mortality rate per country is shown in Fig 3, while the distribution of length of stay and mortality is shown in Figs A and B in S2 Text respectively.

France was chosen as the validation cohort to assess the generalisability among the European cohort because it had the highest number of patients in the database (647, or 22% of the European cohort), with 40% mortality rate (ICU and 30-day) and 19% of patients with a low risk of deterioration. Furthermore, we also evaluated generalisability of the European predictive model on a per-country basis using leave one country out approach. Namely, we selected nine European countries with the highest number of ICU admissions and separately evaluated each corresponding cohort on the model derived from the remaining European countries as shown in S6 Text.

We prospectively evaluated temporal generalisability of our European model using a cohort of 715 (25%) patients admitted to ICU after December 1st, 2020, with a median age of 75 years (IQR, [72–79]), median ICU length of stays 11 days (IQR, [6–20]) and having 32% female patients. The model was derived in a cohort of 2,143 patients with a median age of 75 years (IQR, [72–78]), median ICU length of stay of 14 days (IQR, [8–23]) and having 28% female patients.

Mortality rate in the ICU and 30-day for the derivation cohort (up to cut-off date of December 1^st^, 2020) was 41% and 42% respectively. However, after the cut-off date it increased to 55% and 52% posing a significant challenge for model generalisability. Rate of low-risk patients admitted to ICUs remained at a similar rate of 13% for both cohorts.

**Fig 4:**
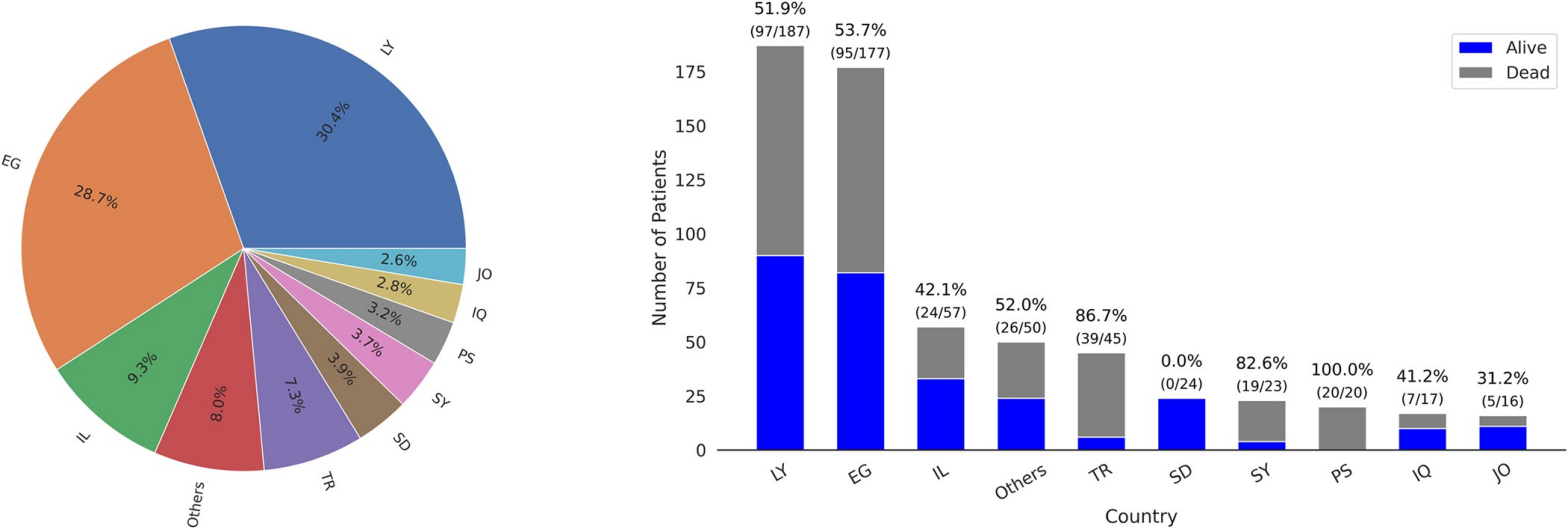
Distribution of patients admitted to non-European ICUs as a percentage of the overall non-European cohort (left panel). Number of patients admitted to non-European ICUs as well as the mortality rate per non-European country, shown as a ratio between survivors (blue) and non-survivors (grey) (right panel). Country and territory abbreviations are detailed in S9 Text.

Finally, the non-European cohort contained 616 patients that had higher ICU and 30-day mortality at 54% in comparison to the European cohort as well as higher rate of low-risk patients admitted to ICU at 25%. Also, the median age of the non-European cohort was 76 years (IQR, [73–81]), with 40% patients female, and the median duration of ICU stay of 7 days (IQR, [4–10]). Detailed information of patient distribution among the non-European countries with a summary of the ICU mortality rate and the number of patients per country are visualised in Fig 4 while the distribution of length of stay and mortality is shown in Figs A and B in S2 Text respectively.

Shown in Table 1 are the detailed characteristics of patients of European and non-European cohorts based on the ICU mortality, while the patient characteristics for the 30-day mortality and low-risk patients are shown in Tables A and B in S7 Text respectively.

**Table 1:** Patient characteristics of the European and the non-European cohort, based on ICU mortality outcome. Patient characteristics for the two other outcomes of interest, namely 30-day mortality and early identification of patients at low risk of deterioration are available in Tables A and B in S7 Text respectively.

ICU	European			non-European		
**Variables**	**Alive**	**Dead**	**p-value**	**Alive**	**Dead**	**p-value**
**Patients (%)**	1574 (0.55)	1284 (0.45)	-	284 (0.46)	332 (0.54)	-
**Age (year)**	74 [72,78]	75 [72,79]	<0.001	75 [72,80]	77 [73,83]	0.003
**Sex (Female)**	477 (30.3)	350 (27.3)	0.081	115 (40.5)	129 (38.9)	0.74
**Weight (kg)**	80 [72,90]	80 [72,90]	0.313	78 [70,89]	78 [70,87]	0.621
**Height (cm)**	170 [165,178]	170 [165,177]	0.162	165 [159,170]	166 [160,173]	0.195
**BMI**	27.6 [24.8,30.9]	27.5 [24.7,30.8]	0.894	27.9 [24.9,33.2]	28.3 [25.6,31.1]	0.737
**SOFA overall score**	4 [3,7]	6 [4,9]	<0.001	4 [2,5]	7 [5,10]	<0.001
**Presence of diabetes**	490 (31.2)	462 (36.2)	0.006	156 (55.1)	187 (56.8)	0.73
**Ischemic heart disease**	318 (20.4)	308 (24.3)	0.015	78 (27.7)	92 (28.6)	0.875
**Renal comorbidity**	193 (12.3)	255 (20.0)	<0.001	29 (10.3)	80 (24.5)	<0.001
**Arterial hypertension**	1029 (65.6)	857 (66.9)	0.484	174 (61.5)	233 (71.3)	0.014
**Pulmonary disease**	341 (21.7)	301 (23.6)	0.241	47 (16.6)	59 (18.4)	0.642
**Congestive heart failure**	203 (13.0)	205 (16.2)	0.019	33 (11.8)	48 (14.8)	0.34
**Mechanical ventilation**	979 (62.2)	1142 (88.9)	<0.001	69 (24.3)	271 (81.6)	<0.001
**Vasopressors**	936 (59.5)	1146 (89.3)	<0.001	38 (13.4)	184 (55.4)	<0.001
**Renal replacement therapy**	150 (9.5)	317 (24.7)	<0.001	16 (5.6)	52 (15.7)	<0.001
**Non-invasive ventilation**	412 (26.2)	340 (26.5)	0.888	74 (26.1)	156 (47.0)	<0.001
**Tracheostomy**	350 (22.2)	241 (18.8)	0.026	15 (5.3)	13 (3.9)	0.537
**ICU length of stay (day)**	12 [6,26]	14 [7,22]	0.097	6 [3.8,10]	7 [4,11]	0.293

### Performance evaluation

**Fig 5:**
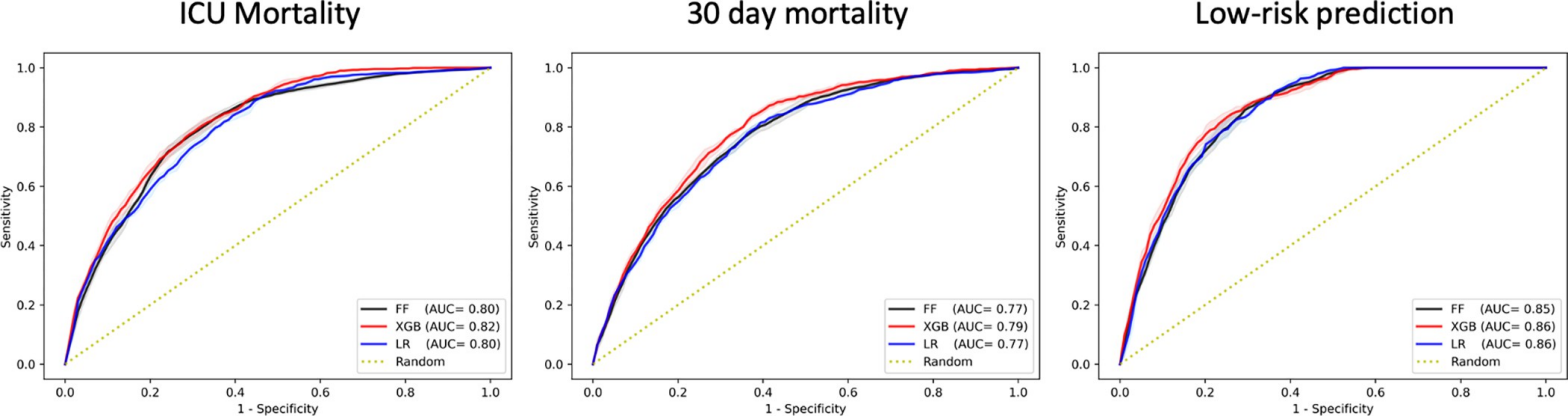
AUC performance of each model in validation on patients admitted in ICUs in France as the country with the highest number of ICU admissions for each of the three outcomes. AUC and AUPRC performance graphs of each model in internal cross-validation are available in Fig A in S3 Text.

All the three algorithms showed similar performance during the evaluation of the model derived in the European cohort (excluding patients admitted to French ICUs) and validated on the French patient cohort. Although, XGBoost had a higher performance with AUC of 0.82 (95% CI 0.82–0.82), 0.79 (95% CI 0.79–0.79) and 0.86 (95% CI 0.86–0.87), for the three outcomes, indicating a high generalisability of the model as shown in Fig 5. XGBoost showed highest performance also in terms of Average Precision (AP), as well as calibration quality (lowest Brier score) shown in Figs D-F in S3 Text. Additional performance metrics including positive and negative predictive value (PPV and NPV), F-1 score and Matthews correlation coefficient (MCC) are shown in S4 Text. Furthermore, we also assessed per country generalisability of our model by evaluating its performance on the nine European countries (with the highest number of ICU admissions) separately, each time deriving the model from the patient cohorts of the remaining countries, in a leave one country out approach. The results of these analyses are shown in Tables A-C in S6 Text for each of the outcomes.

**Fig 6:**
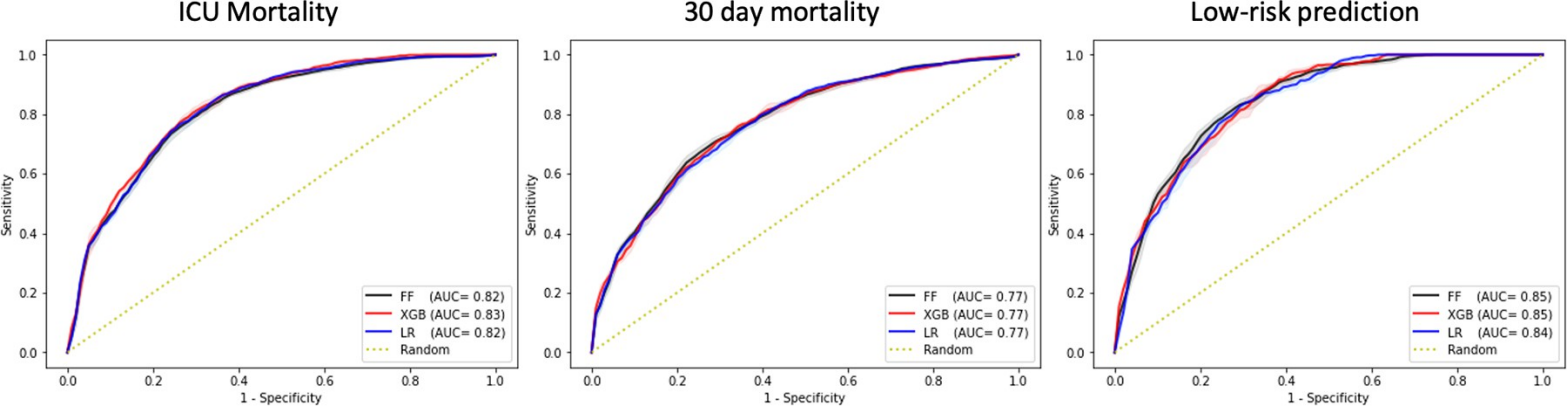
AUC performance of each model in the European cohort of patients admitted to ICUs after the cut-off date of Dec 1st, 2020 for each of the three outcomes. AUC and AUPRC performance graphs of each model in internal cross-validation are available in Fig B in S3 Text.

In the prospective evaluation of the model on the cohort of European patients admitted after the cut-off all three algorithms showed a similar performance in predicting 30-day mortality with AUC of 0.77 (95% CI 0.77–0.77), while XGBoost was superior in predicting ICU mortality with AUC of 0.83 (95% CI 0.83–0.83). In predicting low-risk patients, both FF and XGBoost showed similar performance with AUC of 0.85 (95% CI 0.85–0.85). Performance of each algorithm in terms of AUC is shown in Fig 6, while detailed performance metrics are shown in S4 Text.

**Fig 7:**
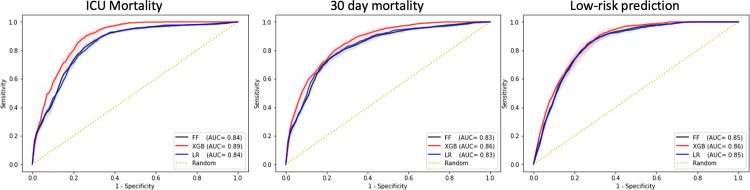
AUC performance of the model derived from the overall European cohort and externally validation in a cohort of Asian, African, and American patients for each of the three outcomes. AUC and AUPRC performance graphs of each model in internal cross-validation are available in Fig C in S3 Text.

Lastly, in the external validation of the European model in a cohort of Asian, African, and American patients, XGBoost achieved AUC of 0.89 (95% CI 0.89–0.89) for ICU mortality, AUC of 0.86 (95% CI 0.86–0.86) for 30-day mortality prediction, and AUC of 0.86 (95% CI 0.86–0.86) in predicting low-risk patients as shown in Fig 7. Furthermore, our results showed that majority of the models are well calibrated as shown from the reliability curves in S3 Text as well as Brier scores in S4 Text.

### Model interpretation and variable importance ranking

This study evaluated three models over three different population study designs with three different outcomes of interest. However, for the analysis of variable importance and model interpretation, we focus on the best performing model, the XGBoost.

**Fig 8:**
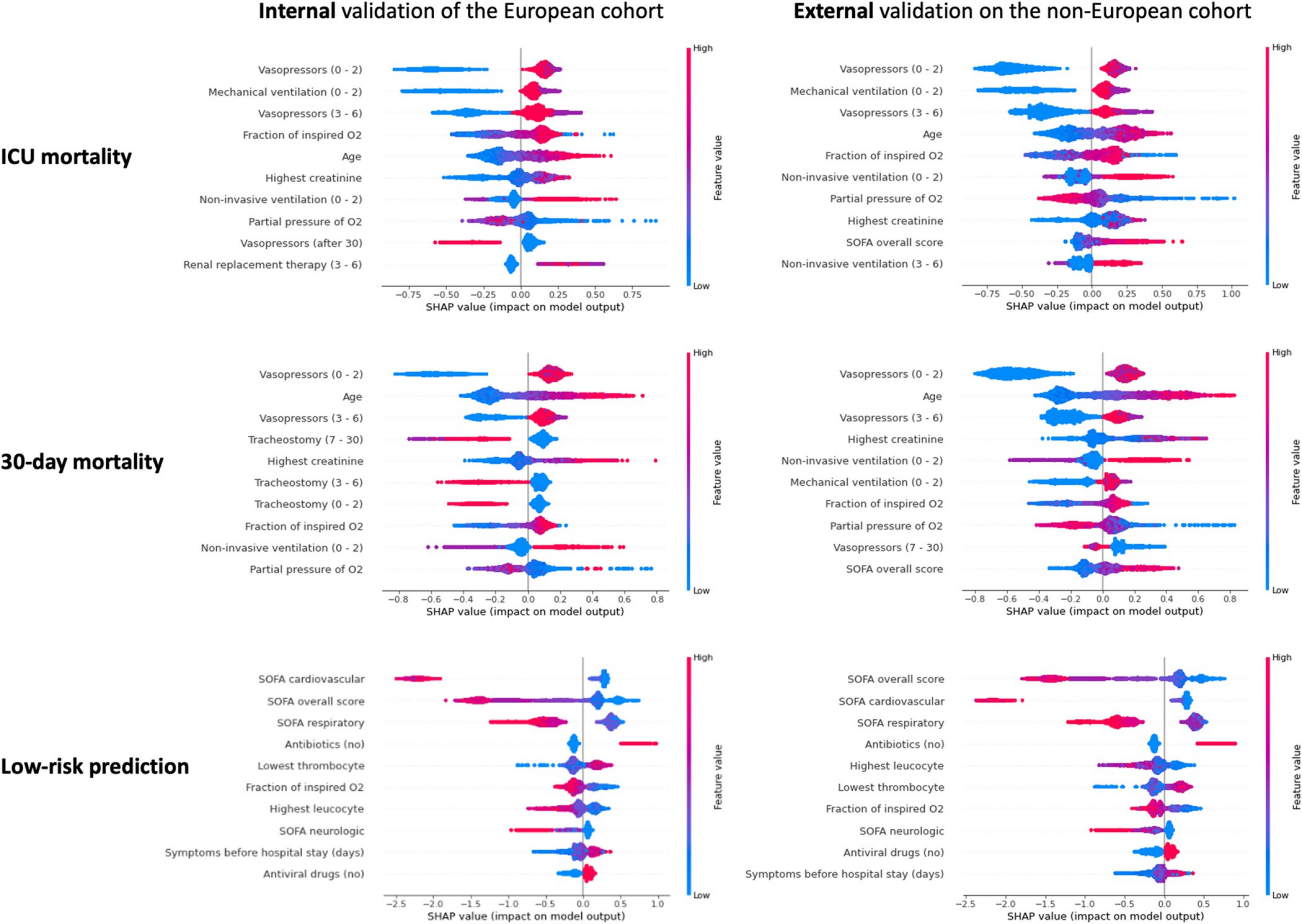
SHAP summary plots showing the top-10 most important variables for each of the three outcomes of interest, when evaluating the European model internally (left) as well as externally on the non-European cohort (right). Colour represents the actual value of the variables (red indicating higher values), while the higher the SHAP value of a variable (depicted in the x-axis), the higher the estimated probability of the outcome. For some variables we also indicate the interval of occurrence, for example “Vasopressors (0–2)” indicates administration of vasopressors during the first 2 days from the admission.

We applied SHAP method over the models’ prediction during each cycle of the 5-fold cross-validation with the 10-times repetition. Unlike the typical approaches in the literature that apply SHAP at the final model only, our approach allows us to investigate predictive impact of each variable much more thoroughly and spot any inconsistencies with the final results. The Beeswarm plots shown in Fig 8 present the relative importance of the top 10 variables and their actual relationships with the predicted outcomes, while the calculated average of absolute SHAP value for each of the ranked variables is available in Fig A in S5 Text.

Vasopressor use and mechanical ventilation within the first 2 days of ICU admission had the highest impact on ICU mortality prediction, followed by the FiO2 and age, for both internal and external cohorts. These two variables were also highly important for the prediction of 30-day mortality in the European and non-European cohorts, while tracheostomy was highly predictive for patients’ survival only in the European cohort. In identifying patients at low risk of mortality, all the 10 highest ranked variables were similar in both the European and the non-European cohorts, with SOFA and its’ sub-scores emerging as the strongest predictive factors.

**Fig 9:**
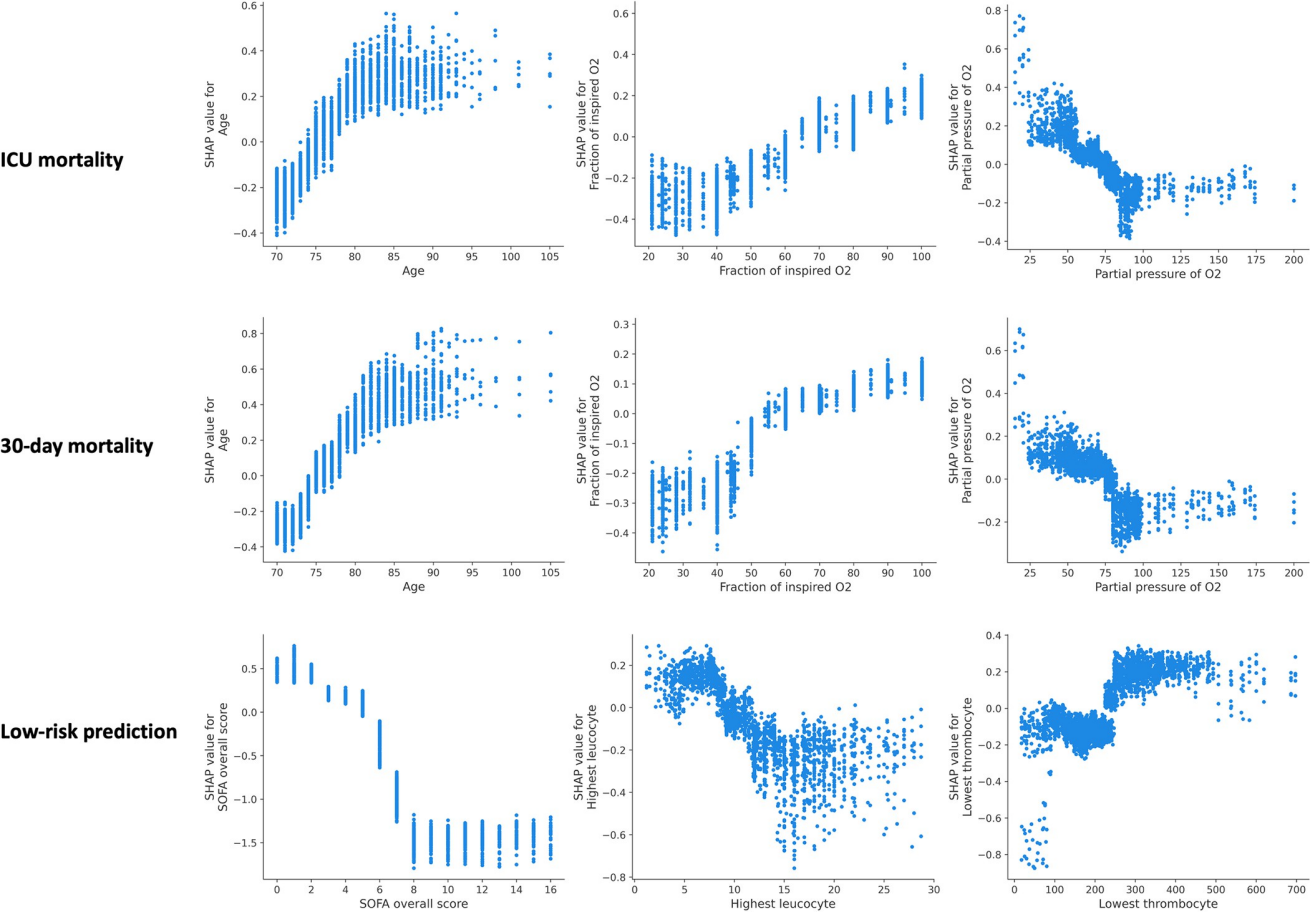
Dependency plots outlining relationship between the actual values of the variables (x-axis) and risk of predicted outcome (y-axis) expressed in terms of SHAP values. Higher SHAP values are associated with an increase in the risk of the outcome and vice-versa. For example, increasing age (x-axis in the top left graph), gradually increases the risk of predicted ICU mortality up to around 80 years. Beyond this value, the risk remains high. These results pertain to the model derived on the overall European cohort and validated externally on the non-European cohort. Values of FiO2 are expressed in percentages and PaO2 in mmHg, while leucocytes and thrombocytes are in million per microliter (x 10^9^/μL).

In addition to the importance of the overall variables, we sought to also investigate the values of individual continuous variables and their association to the predicted risk of outcome. From these analyses, shown in Fig 9, the predicted risk of ICU mortality gradually increases with age until around 80 years, beyond which remains high. Predicted 30-day mortality shows a similar pattern, although the age threshold appears to be slightly higher, at around 85. FiO2 values of up to 40% do not appear to increase the predicted risk of ICU and 30-day mortality, while PaO2 values of 75 mmHg or lower are associated with a sharp increase in the predicted risk of ICU and 30-day mortality. Lastly, increase in SOFA scores, gradually augment the predicted risk of deterioration (as would be expected), however only up to a threshold value of 8, where beyond these SOFA scores the predicted risk remains consistently high. Very low values of leucocytes (below 4.5 x 10^9^/μL) and those above 11 x 10^9^/μL appear to increase the predicted risk of deterioration, however the picture is less clear cut for values beyond this range as there is a high variability between the patients. Low values of thrombocytes, below 100 x 10^9^/μL appear to increase the predicted risk of deterioration, but only in a fraction of the patients, while the predicted risk appears to be decrease above this value.

**Fig 10:**
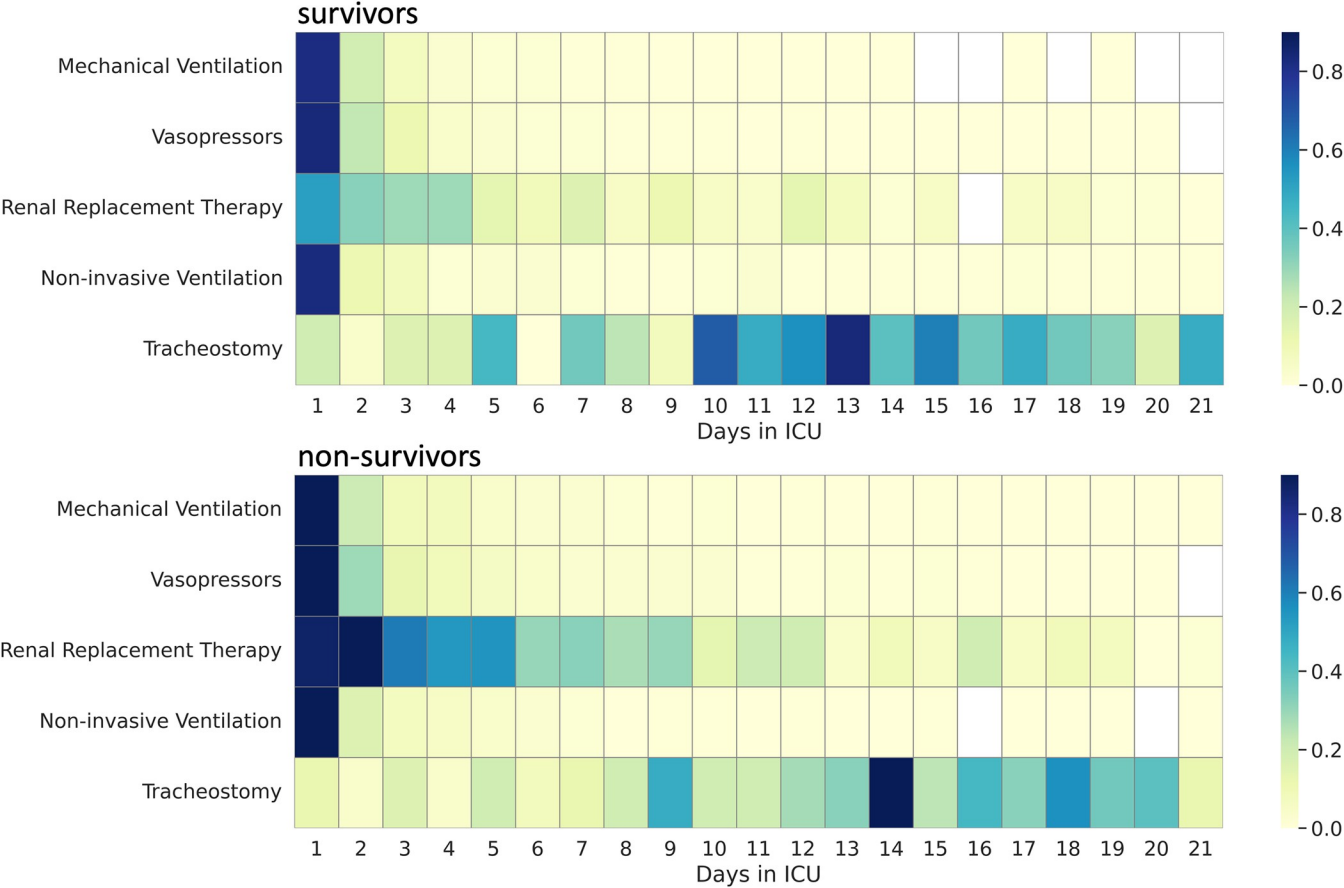
Differences between patients that survived in the ICU and those that did not, in terms of frequency of interventions administered in a particular day during their ICU stay, in the European cohort. Shade of colour represents the frequency of administration for the overall European cohort. The darker the shade, the more patients were administered a particular intervention on that specific day. White boxes represent no recorded interventions for that day.

Furthermore, considering that the starting day of intervention had a significant impact on the predicted mortality, we sought to investigate the differences between the patients that survived in the ICU and those that did not, in terms of days when various interventions were administered during the ICU stay. As can be seen from Fig 10 patients that did not survive had Renal Replacement Therapy administered more often during the first week of stay (bottom graph, dark blue) than the patients that survived (top graph, light blue). Furthermore, for patients that survived, tracheostomy was administered more often in comparison to patients that did not survive. In terms of the other types of interventions, namely mechanical and non-invasive ventilation as well as vasopressors, we did not find significant differences in our dataset.

## Discussion

This is one of the first studies to demonstrate high performance generalisability (AUC up to 0.86) of machine learning models in predicting clinically relevant outcomes of older patients from diverse patient populations with COVID-19, including patients from different European countries, across different continents and ethnicities, as well as patients admitted in different COVID-19 waves.

While there have been several previous studies that have investigated feasibility of machine learning for predicting deterioration and mortality of COVID-19 patients [10–13] including resource allocation [14], none of the studies have evaluated generalisability across highly geographically diverse patient populations [7].

Furthermore, this level of generalisability performance was achieved using only routinely collected clinical and demographic variables, suggesting the applicability of our method also in low-end equipped ICUs and healthcare institutions.

Achieving high generalisability with diverse patient populations is important since availability of this type of model, especially in countries with limited resources and expertise could become an important decision-making aid, lending objective support to the complex issue of resource allocation. These models might prove particularly important in patients, where the best course of therapeutic action is difficult to judge at the admission due to disease complexity or lack of prior expertise when facing a novel disease.

To further aid decision making we performed saliency analysis on our model such that clinicians can identify patients at low risk of deterioration, and consequently the care and resources can be prioritised as early as possible. Our findings are consistent with previous studies highlighting importance of clinical markers such as age and oxygen saturation. However, while we find that SOFA is a good predictor of estimating patients at low risk, it becomes less important in mortality prediction, which our model estimates principally through age, and FiO2 variables. This finding is in line with the previous work, which found poor discriminant accuracy of the SOFA score for mortality prediction [6,24].

Our model also captured the association between increased risk of mortality and administration of vasopressors and mechanical ventilation within the first 48 hours after the admission, as shown in Fig 8. In the same line, performing tracheostomy after the first week of admission increased the risk of 30-day mortality, but not of ICU mortality.

While patients’ age increases the risk of mortality, this appears true only until a threshold of 80 years, after which the risk of ICU mortality remains high. Similarly values of FiO2 up to 40% do not appear to increase the risk of estimated ICU and 30-day mortality, while an increase in SOFA scores increase the estimated risk of deterioration, but only up to the score of 8. Beyond this SOFA score the risk remains high, as shown in Fig 9. Finally, very low values of leucocyte count (below 4 x 10^9^/μL) increase the estimated risk of deterioration and the same is true for low values of thrombocytes (below 100 x 10^9^/μL), but only in a fraction of the patients for the latter.

Majority of the previous studies utilised only admission data to predict the risk of a single event, while typically not considering the subsequent therapeutical interventions, except for the work in [25] that focused on prediction of favourable outcomes and in [26] that focused on predicting the need for mechanical ventilation, validating their models within a patient population from a single country. One of the advantages of our methodology is that our predictive model can generate continuous risk prediction scores, taking into consideration also therapeutical interventions, such as vasopressors or mechanical ventilation, in updating risk estimation. Moreover, we have shown that the continuous risk estimation can be applied to highly diverse patient populations.

Many design and implementation decisions of our work have been made with a future clinical practice deployment in mind. In this respect, geographical and temporal evaluation of the model as well as continuous risk prediction would be important steps in understanding performance of the model in a clinical practice. Furthermore, our models are not only interpretable in terms of importance of variables based on SHAP values, but we also provide specific cut-off points for some of the variables where the risk of outcome increases significantly, building upon our previous work [24]

These results show that our model derived from a cohort of European patients can be used to predict outcomes of interest in patients admitted to non-European ICUs, rendering it particularly relevant for countries where essential resources (such as ventilators) might be scarce, with varying availability of clinical expertise. From this analysis we believe that our model can support physicians in estimating prognosis and therapy course. However, this model should be seen as an additional tool that supports clinical decision making as part of a holistic patient assessment, while the final decision rests with the judgement of the clinicians, especially considering ethical issues [27,28].

### Limitations

The present study has some methodological limitations in common with the other COVIP-studies [29–33], such as COVIP does not contain a control group of younger COVID-19 patients for comparison, or a comparable age cohort of ICU / non-ICU patients. In addition, the COVIP database does record information on time from symptoms onset to ICU admission. These treatment limitations might also affect the care of older ICU patients. Furthermore, COVIP recruited patients in many countries with a wide variety in their care structure, resulting in a considerable heterogeneity of treatments.

## Conclusions

This study demonstrates that even in the case of very diverse COVID-19 patients from other countries and continents, machine learning methods can generalise well and produce precise risk estimates to support clinical decision making. Our models captured both the dynamic course of the disease by including occurrence and time-to-event information of clinical events as well as similarities and differences between the diverse cohorts, allowing prediction of disease severity, identification of low-risk patients and potentially supporting effective planning of essential intensive care resources.

## Supporting information

S1 Text Definition of comorbidities. (DOCX)

S2 Text Distribution of length of stay and mortality for the European and non-European cohorts as well as difference between COVID-19 waves. (DOCX)

S3 Text Performance for Area Under the ROC curve, Precision Recall curve as well as model calibration analysis for internal, prospective, and external validation cohorts for each of the outcomes of interest. (DOCX)

S4 Text Detailed performance metrics, including Average Precision, Positive and Negative Predictive Value, F-1 score, Mathews Correlation Coefficient as well as Brier calibration score, for the internal, prospective, and the external validation cohorts for each of the outcomes of interest. (DOCX)

S5 Text Variable ranking for the European and non-European cohort for each of the three outcomes of interest. (DOCX)

S6 Text Evaluation of generalisability of the model derived on the European cohort using each of the top-9 European countries (based on the number of ICU admissions) as the test cohort. (DOCX)

S7 Text Patients’ characteristics, including differences between the full set of variables, for the European and non-European cohort with respect to the three outcomes of interest (ICU and 30-day mortality and prediction of low-risk patients). (DOCX)

S8 Text Cohort selection diagram. (DOCX)

S9 Text Abbreviations of the countries and territories. (DOCX)
