# Supplementary material for: COVID-19 machine learning model predicts outcomes in older patients from various European countries, between pandemic waves, and in a cohort of Asian, African, and American patients
Source: PLOS Digit Health. 2022 Nov 8;1(11):e0000136. doi: 10.1371/journal.pdig.0000136 (PMC9931233; doi:10.1371/journal.pdig.0000136)
Supplement: S1 Text — (DOCX) [file pdig.0000136.s001.docx]

S1 Text – Definition of comorbidities

**Diabetes mellitus:** documented evidence of diabetes mellitus or reported by the patient or their relatives. Prescription of anti-diabetic medication or insulin on the drug chart.

**Ischaemic heart disease:** documented abnormal coronary angiography, known coronary artery disease, previous percutaneous coronary intervention (PCI) or coronary bypass surgery

**Chronic renal failure:** documented evidence of chronic renal insufficiency Grade 3 or higher, creatinine clearance <60ml/min or chronic dialysis

**Arterial hypertension:** documented evidence of any grade of chronic arterial hypertension or prescription of anti-hypertensive medication.

**Pulmonary disease:** documented evidence of or medication prescribed for chronic pulmonary disease of any aetiology (bronchial asthma, COPD, pulmonary fibrosis), or clinical or radiological signs of chronic pulmonary disease

**Chronic heart failure:** documented evidence of or medication prescribed for chronic heart failure of any aetiology or echocardiographic or radiological signs of chronic heart failure.
