# Supplementary material for: COVID-19 machine learning model predicts outcomes in older patients from various European countries, between pandemic waves, and in a cohort of Asian, African, and American patients
Source: PLOS Digit Health. 2022 Nov 8;1(11):e0000136. doi: 10.1371/journal.pdig.0000136 (PMC9931233; doi:10.1371/journal.pdig.0000136)
Supplement: S2 Text — (DOCX) [file pdig.0000136.s002.docx]

# S2 Text – Distribution of length of stay and mortality for the European and non-European cohorts as well as difference between COVID-19 waves

| 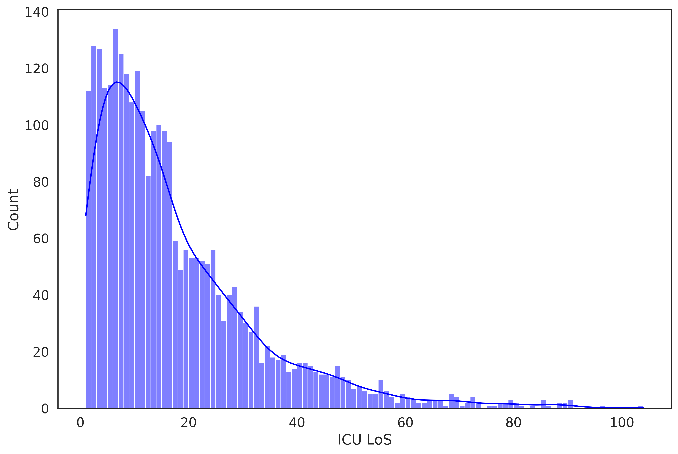 | 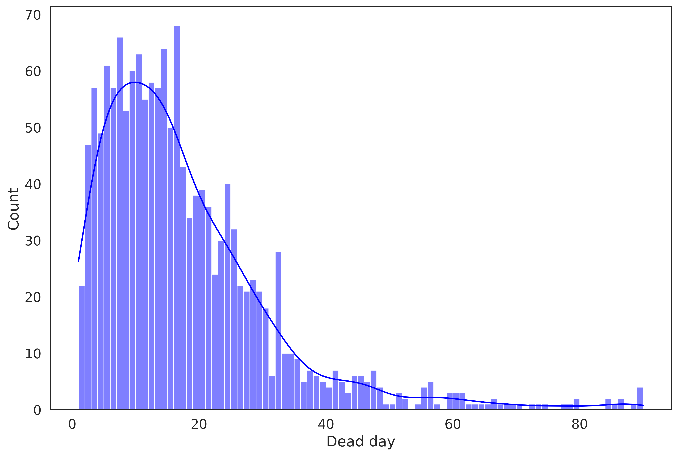 |
| --- | --- |

Fig A Distribution of length of stay (LoS) and mortality rate (in days) for the European cohort, in the left and right panel respectively.

| 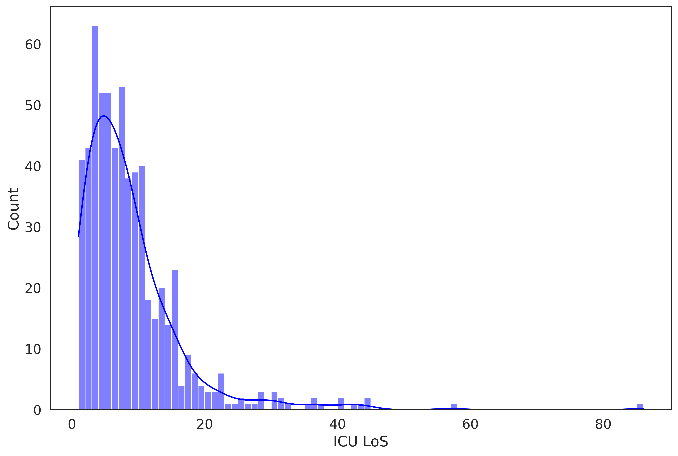 | 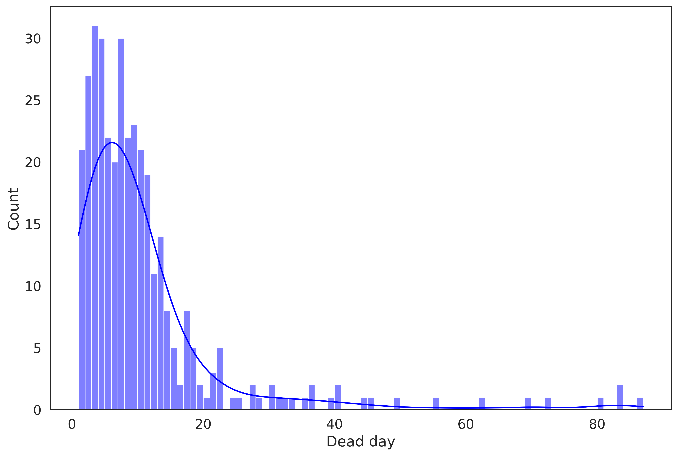 |
| --- | --- |

Fig B Distribution of length of stay (LoS) and mortality rate (in days) for the non-European cohort, in the left and right panel respectively.


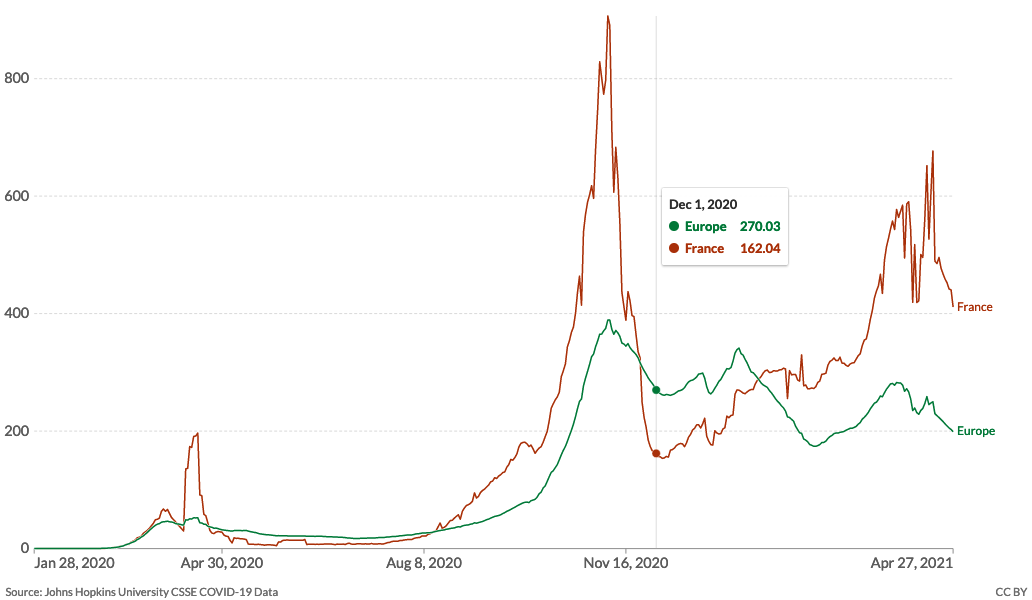


Fig C Number of daily confirmed cases per million people, between the first two COVID-19 waves in Europe and France (country with the highest number of ICU admissions in our dataset) from January 28^th^, 2020 (earliest recorded data in the source) up to April 27^th^, 2020 (the end date in our dataset). The first wave peaked around November 7^th^, 2020, and subsided around Dec 1^st^, 2020, which was our cut-off date between the first two waves, to allow us to evaluate the model derived on the patient cohort during the first wave using the patient cohort of the second wave. (Source Our World in Data, John Hopkins University).
