# Supplementary material for: COVID-19 machine learning model predicts outcomes in older patients from various European countries, between pandemic waves, and in a cohort of Asian, African, and American patients
Source: PLOS Digit Health. 2022 Nov 8;1(11):e0000136. doi: 10.1371/journal.pdig.0000136 (PMC9931233; doi:10.1371/journal.pdig.0000136)
Supplement: S3 Text — (DOCX) [file pdig.0000136.s003.docx]

# S3 Text – Performance for Area Under the ROC curve, Precision Recall curve as well as model calibration analysis for internal, prospective, and external validation cohorts for each of the outcomes of interest

**
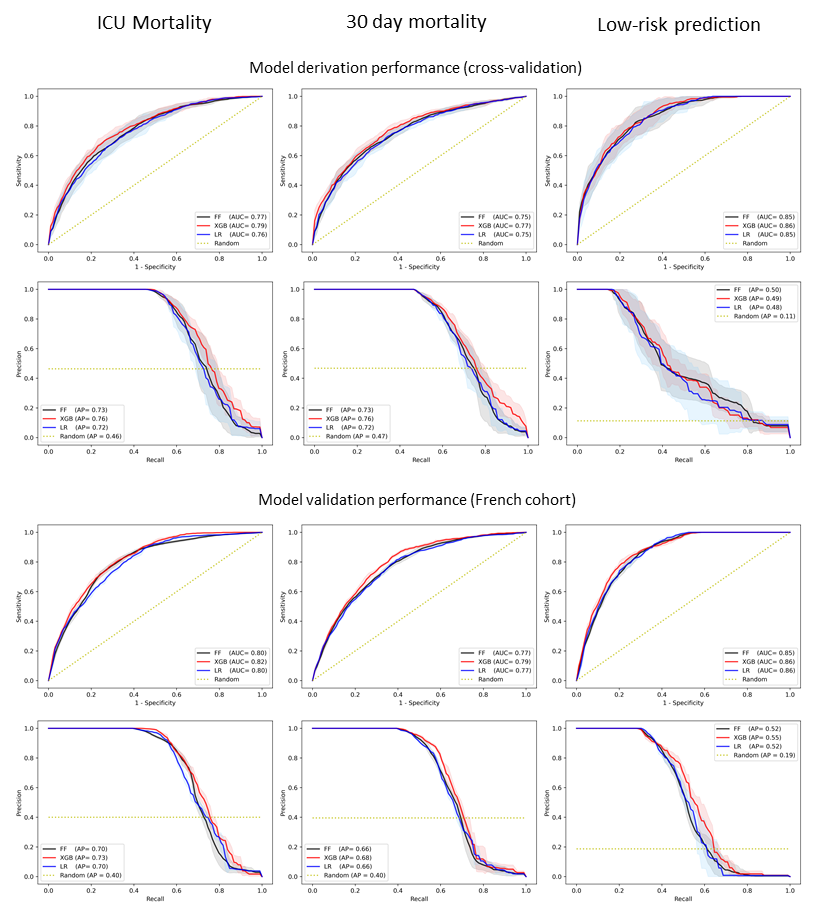
**

*Fig A Retrospective AUC (top row) and AUPRC (bottom row) performance of each model in internal cross-validation (top panel) and external validation on patients admitted in ICUs in France as the country with the highest number of ICU admissions (bottom panel) for each of the three outcomes.*


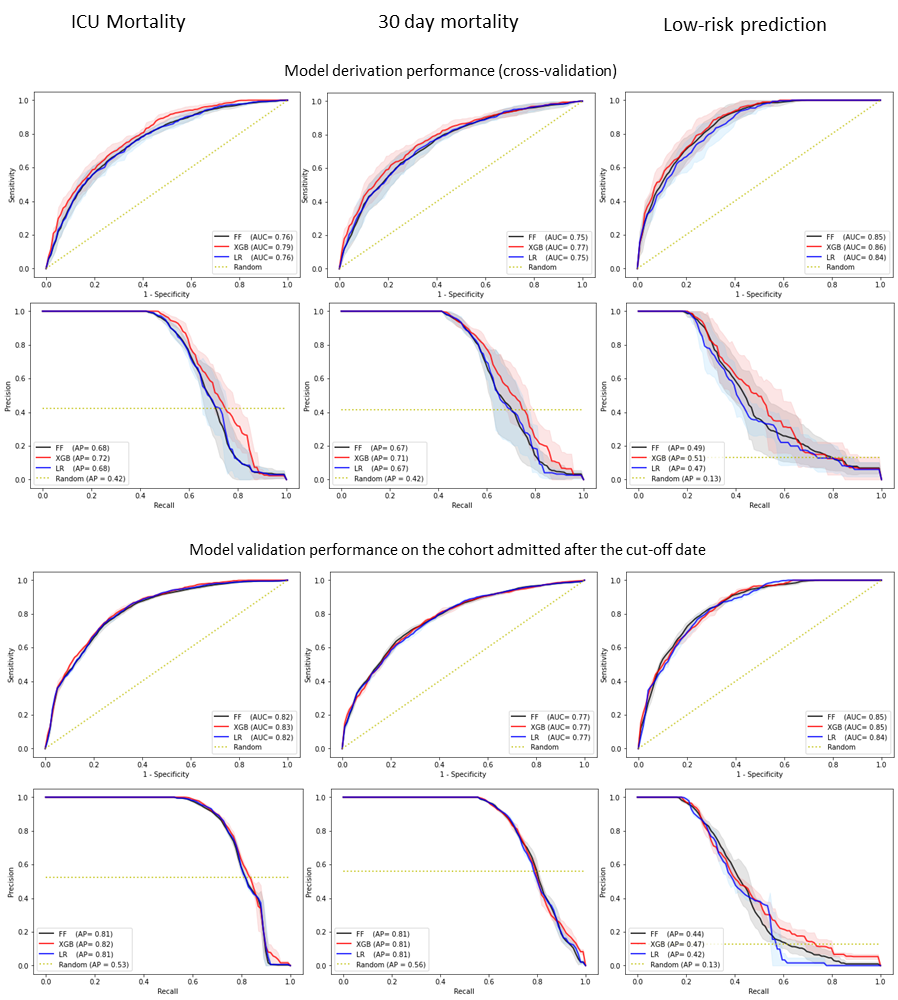


*Fig B Prospective AUC (top row) and AUPRC (bottom row) performance of each model in internal cross-validation (top) of the cohort of patients admitted to ICUs from January 11, 2020, until the cut-off date of December 1^st^, 2020 (top). Prospective validation on the cohort of patients admitted to ICUs after the cut-off date until April 27, 2021 (bottom) for each of the three outcomes.*


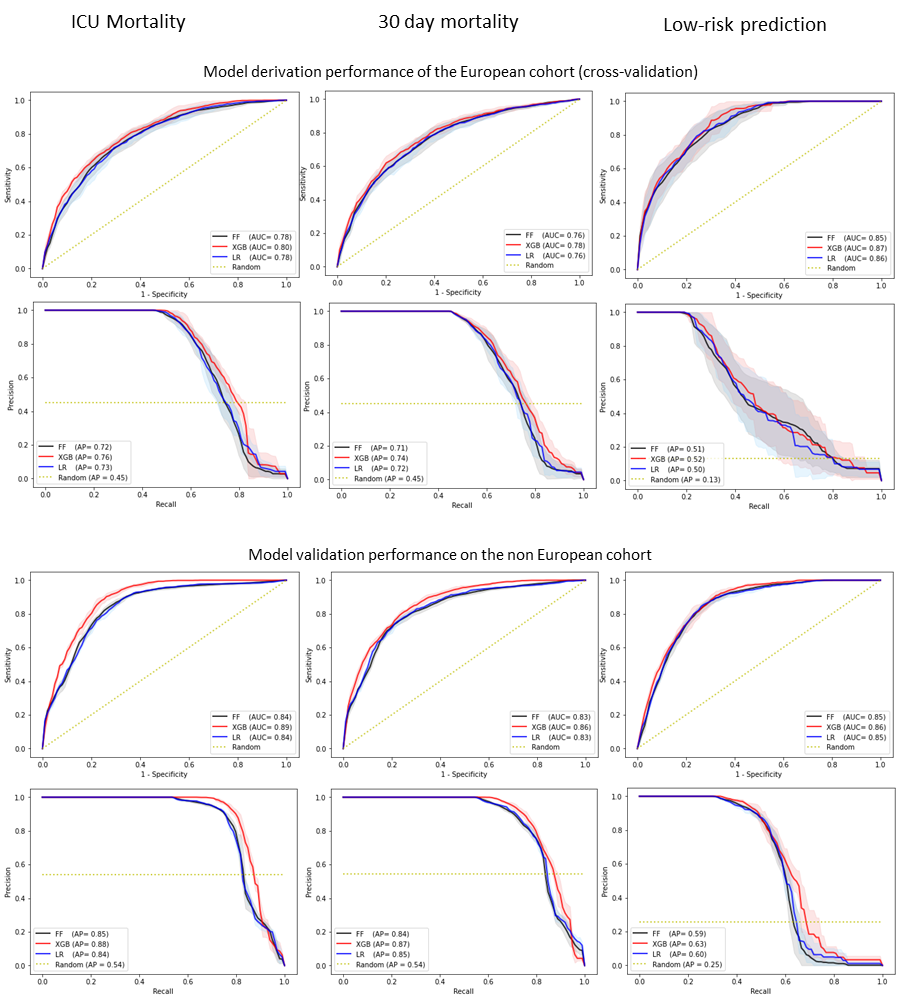


*Fig C AUC (top row) and AUPRC (bottom row) performance of cross validation of the model derived on the overall European cohort (top panel) and its external validation in a cohort of Asian, African and American patients (bottom panel)for each of the three outcomes.*

*
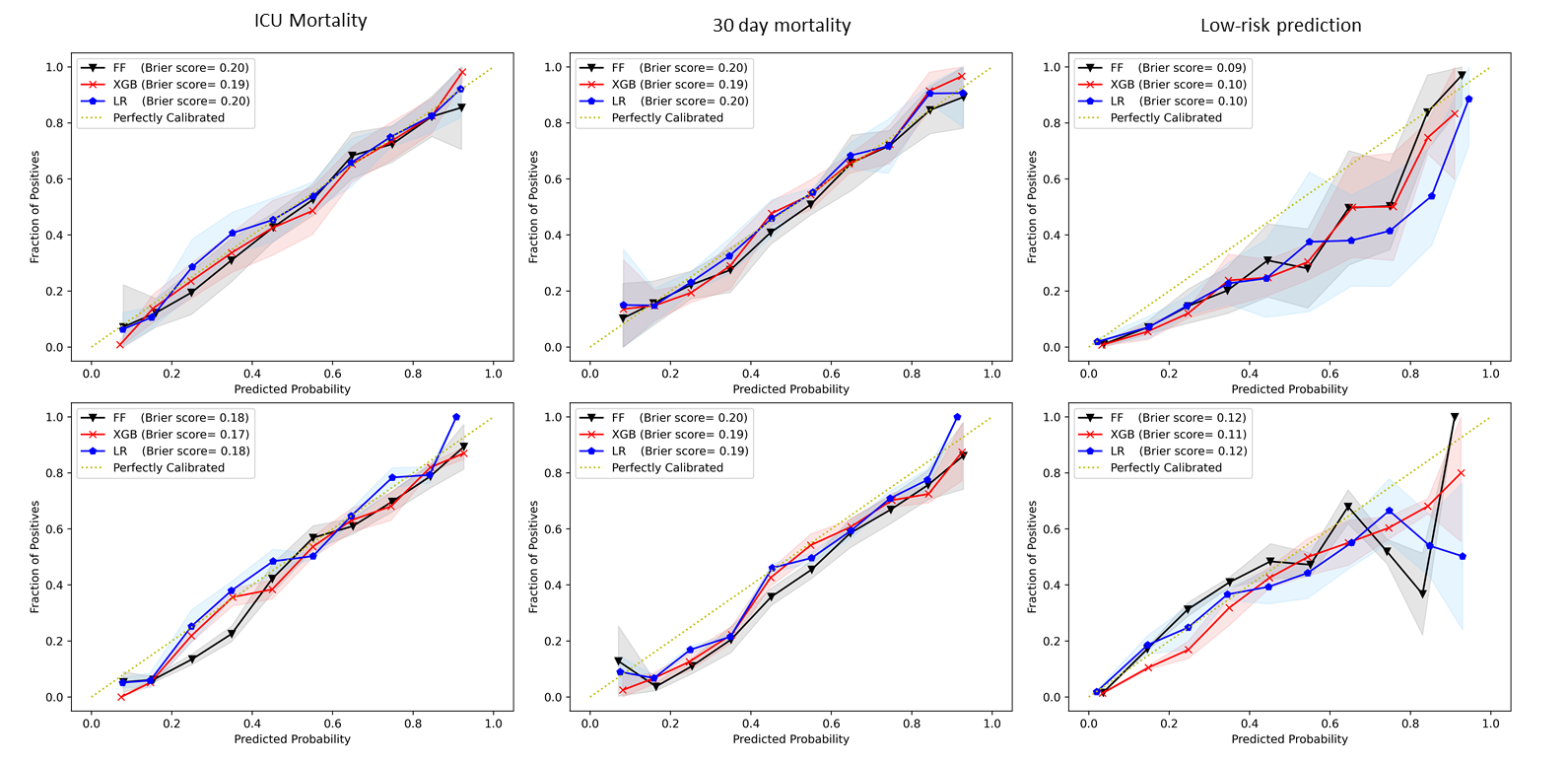
*

*Fig D Calibration quality of each model in internal cross-validation (top) and external validation on France as the country with the highest number of ICU admissions (bottom) for each of the three outcomes.*

***
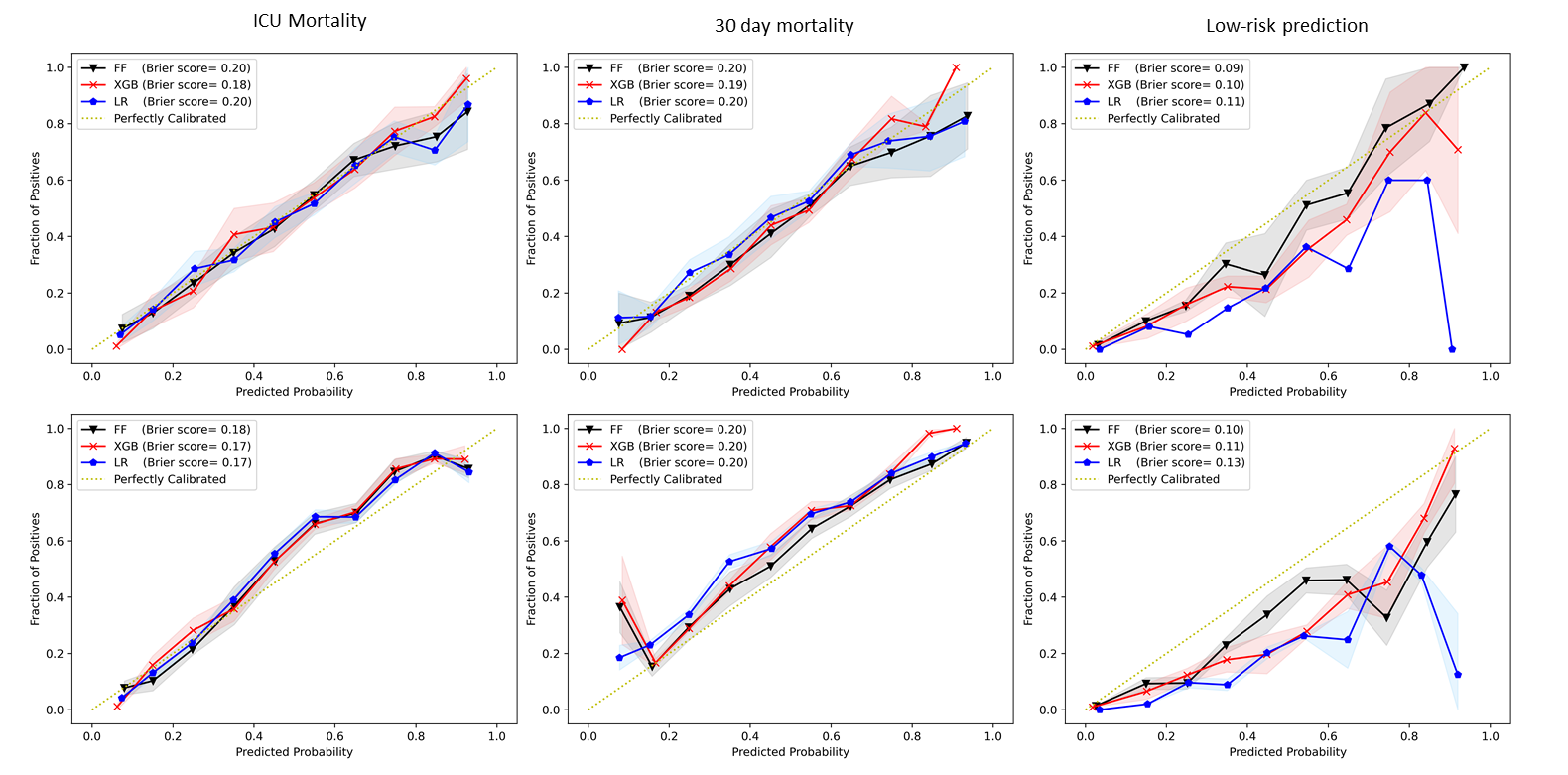
***

*Fig E Calibration quality of each model in internal cross-validation (top) of the cohort of patients admitted to ICUs from January 11, 2020, until the cut-off date of December 1st, 2020 (top). Prospective validation on the cohort of patients admitted to ICUs after the cut-off date until April 27, 2021 (bottom) for each of the three outcomes.*

**
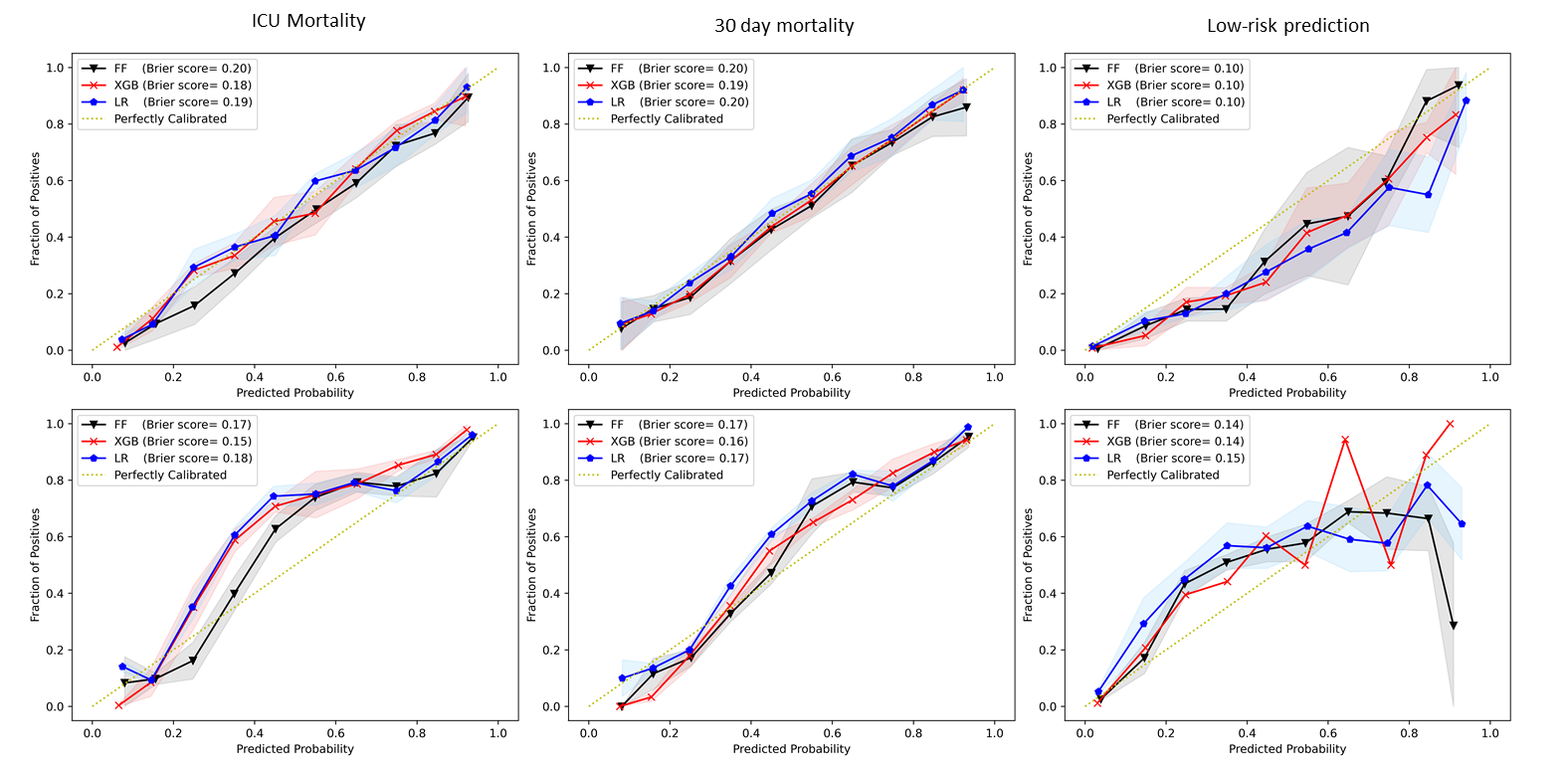
**

*Fig F Calibration quality of cross validation of the overall European cohort (top) and external validation of the model in a cohort of Asian, African and American patients (bottom) for each of the three outcomes.*
