# Supplementary material for: COVID-19 machine learning model predicts outcomes in older patients from various European countries, between pandemic waves, and in a cohort of Asian, African, and American patients
Source: PLOS Digit Health. 2022 Nov 8;1(11):e0000136. doi: 10.1371/journal.pdig.0000136 (PMC9931233; doi:10.1371/journal.pdig.0000136)
Supplement: S4 Text — (DOCX) [file pdig.0000136.s004.docx]

# S4 Text - Detailed performance metrics, including Average Precision, Positive and Negative Predictive Value, F-1 score, Mathews Correlation Coefficient as well as Brier calibration score, for the internal, prospective, and the external validation cohorts for each of the outcomes of interest

## Retrospective evaluation of European cohort

| ***ICU*** | **AUC** | **AP** | **PPV** | **NPV** | **F-1** | **MCC** | **Brier** |
| --- | --- | --- | --- | --- | --- | --- | --- |
| **LR** | 0.76  [0.76-0.77] | 0.72  [0.71-0.73] | 0.67  [0.65-0.68] | 0.71  [0.70-0.71] | 0.66  [0.65-0.67] | 0.37  [0.36-0.39] | 0.20  [0.19-0.20] |
| **FF** | 0.77  [0.76-0.78] | 0.72  [0.71-0.73] | 0.66  [0.65-0.67] | **0.73**  [0.72-0.74] | **0.68**  [0.67-0.69] | 0.39  [0.38-0.41] | 0.20  [0.20-0.20] |
| **Xgb** | **0.79**  [0.78-0.79] | **0.76**  [0.75-0.76] | **0.68**  [0.67-0.69] | **0.73**  [0.73-0.74] | **0.68**  [0.68-0.69] | **0.41**  [0.40-0.42] | **0.19**  [0.19-0.19] |

| ***30-day*** | **AUC** | **AP** | **PPV** | **NPV** | **F-1** | **MCC** | **Brier** |
| --- | --- | --- | --- | --- | --- | --- | --- |
| **LR** | 0.75  [0.74-0.76] | 0.72  [0.71-0.73] | 0.67  [0.66-0.68] | 0.70  [0.70-0.70] | 0.66  [0.65-0.66] | 0.37  [0.36-0.37] | 0.20  [0.20-0.21] |
| **FF** | 0.75  [0.75-0.76] | 0.72  [0.71-0.73] | 0.66  [0.65-0.67] | **0.72**  [0.72-0.73] | **0.68**  [0.67-0.68] | 0.38  [0.37-0.39] | 0.20  [0.20-0.21] |
| **Xgb** | **0.77**  [0.77-0.78] | **0.76**  [0.75-0.76] | **0.69**  [0.68-0.70] | 0.71  [0.71-0.72] | 0.67  [0.67-0.68] | **0.40**  [0.38-0.41] | **0.19**  [0.19-0.20] |

| ***Low risk*** | **AUC** | **AP** | **PPV** | **NPV** | **F-1** | **MCC** | **Brier** |
| --- | --- | --- | --- | --- | --- | --- | --- |
| **LR** | 0.85  [0.84-0.86] | 0.48  [0.46-0.50] | 0.44  [0.39-0.49] | 0.92  [0.92-0.92] | 0.43  [0.41-0.45] | 0.36  [0.34-0.39] | 0.10  [0.09-0.10] |
| **FF** | 0.85  [0.84-0.87] | **0.50**  [0.48-0.52] | **0.51**  [0.48-0.54] | 0.92  [0.92-0.92] | 0.42  [0.40-0.44] | 0.37  [0.35-0.39] | **0.09**  [0.08-0.09] |
| **Xgb** | **0.86**  [0.85-0.87] | 0.49  [0.47-0.51] | 0.44  [0.42-0.47] | **0.93**  [0.93-0.93] | **0.44**  [0.43-0.45] | **0.37**  [0.36-0.38] | 0.10  [0.09-0.10] |

## External validation on the French cohort

| ***ICU*** | **AUC** | **AP** | **PPV** | **NPV** | **F-1** | **MCC** | **Brier** |
| --- | --- | --- | --- | --- | --- | --- | --- |
| **LR** | 0.80  [0.80-0.80] | 0.70  [0.70-0.70] | 0.65  [0.64-0.65] | 0.75  [0.75-0.76] | 0.63  [0.63-0.64] | 0.40  [0.39-0.40] | 0.18  [0.18-0.18] |
| **FF** | 0.80  [0.80-0.80] | 0.70  [0.70-0.71] | 0.66  [0.66-0.67] | 0.79  [0.79-0.80] | 0.68  [0.68-0.69] | 0.46  [0.46-0.47] | 0.18  [0.18-0.18] |
| **Xgb** | **0.82**  [0.82-0.82] | **0.73**  [0.72-0.73] | **0.67**  [0.66-0.67] | **0.80**  [0.80-0.80] | **0.69**  [0.69-0.69] | **0.47**  [0.47-0.48] | **0.17**  [0.17-0.17] |

| ***30-day*** | **AUC** | **AP** | **PPV** | **NPV** | **F-1** | **MCC** | **Brier** |
| --- | --- | --- | --- | --- | --- | --- | --- |
| **LR** | 0.77  [0.76-0.77] | 0.66  [0.66-0.66] | 0.60  [0.60-0.60] | 0.76  [0.76-0.77] | 0.63  [0.63-0.63] | 0.37  [0.37-0.37] | **0.19**  [0.19-0.20] |
| **FF** | 0.77  [0.77-0.77] | 0.66  [0.66-0.66] | 0.59  [0.59-0.60] | **0.79**  [0.79-0.80] | 0.65  [0.65-0.66] | 0.39  [0.39-0.40] | 0.20  [0.20-0.20] |
| **Xgb** | **0.79**  [0.79-0.79] | **0.68**  [0.68-0.68] | **0.64**  [0.63-0.64] | **0.79**  [0.79-0.79] | **0.67**  [0.67-0.67] | **0.44**  [0.43-0.44] | **0.19**  [0.19-0.19] |

| ***Low risk*** | **AUC** | **AP** | **PPV** | **NPV** | **F-1** | **MCC** | **Brier** |
| --- | --- | --- | --- | --- | --- | --- | --- |
| **LR** | **0.86**  [0.85-0.86] | 0.50  [0.50-0.51] | 0.55  [0.54-0.55] | 0.87  [0.87-0.87] | 0.48  [0.47-0.49] | 0.38  [0.38-0.39] | 0.12  [0.12-0.12] |
| **FF** | 0.85  [0.85-0.85] | 0.51  [0.50-0.51] | 0.55  [0.55-0.56] | 0.85  [0.85-0.86] | 0.39  [0.38-0.39] | 0.31  [0.31-0.32] | 0.12  [0.12-0.12] |
| **Xgb** | **0.86**  [0.86-0.87] | **0.54**  [0.54-0.55] | **0.57**  [0.56-0.57] | **0.88**  [0.88-0.88] | **0.50**  [0.50-0.51] | **0.41**  [0.40-0.42] | **0.11**  [0.11-0.11] |

## Prospective evaluation performance of the European cohort in internal cross-validation

| ***ICU*** | **AUC** | **AP** | **PPV** | **NPV** | **F-1** | **MCC** | **Brier** |
| --- | --- | --- | --- | --- | --- | --- | --- |
| **LR** | 0.76  [0.76-0.77] | 0.68  [0.67-0.69] | 0.64  [0.64-0.65] | 0.74  [0.73-0.74] | 0.64  [0.64-0.65] | 0.38  [0.37-0.39] | 0.20  [0.19-0.20] |
| **FF** | 0.76  [0.76-0.77] | 0.68  [0.67-0.69] | 0.65  [0.64-0.66] | 0.73  [0.73-0.74] | 0.64  [0.63-0.65] | 0.38  [0.37-0.39] | 0.20  [0.19-0.20] |
| **Xgb** | **0.79**  [0.79-0.80] | **0.72**  [0.71-0.72] | **0.67**  [0.66-0.68] | **0.75**  [0.74-0.75] | **0.66**  [0.65-0.66] | **0.41**  [0.40-0.43] | **0.18**  [0.18-0.18] |

| ***30-day*** | **AUC** | **AP** | **PPV** | **NPV** | **F-1** | **MCC** | **Brier** |
| --- | --- | --- | --- | --- | --- | --- | --- |
| **LR** | 0.75  [0.75-0.76] | 0.67  [0.66-0.68] | 0.65  [0.64-0.67] | 0.72  [0.71-0.73] | 0.61  [0.60-0.62] | 0.37  [0.35-0.39] | 0.20  [0.20-0.20] |
| **FF** | 0.75  [0.75-0.76] | 0.67  [0.66-0.68] | 0.63  [0.62-0.64] | **0.74**  [0.74-0.75] | 0.64  [0.63-0.65] | 0.38  [0.36-0.39] | 0.20  [0.20-0.20] |
| **Xgb** | **0.77**  [0.77-0.78] | **0.70**  [0.69-0.72] | **0.66**  [0.65-0.67] | **0.74**  [0.73-0.75] | **0.63**  [0.62-0.64] | **0.39**  [0.37-0.41] | **0.19**  [0.19-0.19] |

| ***Low risk*** | **AUC** | **AP** | **PPV** | **NPV** | **F-1** | **MCC** | **Brier** |
| --- | --- | --- | --- | --- | --- | --- | --- |
| **LR** | 0.84  [0.83-0.85] | 0.47  [0.45-0.48] | 0.45  [0.43-0.46] | 0.90  [0.90-0.90] | 0.42  [0.42-0.43] | 0.34  [0.33-0.35] | 0.11  [0.11-0.11] |
| **FF** | 0.85  [0.85-0.86] | 0.49  [0.47-0.51] | **0.64**  [0.60-0.68] | 0.90  [0.90-0.90] | 0.36  [0.34-0.39] | 0.35  [0.33-0.37] | **0.09**  [0.09-0.09] |
| **Xgb** | **0.87**  [0.86-0.87] | **0.51**  [0.49-0.53] | 0.47  [0.45-0.50] | **0.92**  [0.92-0.92] | **0.48**  [0.46-0.49] | **0.40**  [0.38-0.42] | 0.10  [0.09-0.10] |

## Prospective evaluation on the cohort of patients admitted after the cut-off date

| ***ICU*** | **AUC** | **AP** | **PPV** | **NPV** | **F-1** | **MCC** | **Brier** |
| --- | --- | --- | --- | --- | --- | --- | --- |
| **LR** | 0.82  [0.82-0.82] | 0.81  [0.81-0.81] | 0.77  [0.77-0.77] | 0.72  [0.72-0.72] | 0.75  [0.75-0.75] | 0.49  [0.49-0.50] | **0.17**  [0.17-0.17] |
| **FF** | 0.82  [0.82-0.82] | 0.81  [0.81-0.81] | 0.77  [0.77-0.77] | 0.72  [0.71-0.72] | 0.75  [0.74-0.75] | 0.48  [0.48-0.49] | 0.18  [0.18-0.18] |
| **Xgb** | **0.83**  [0.83-0.83] | **0.82**  [0.82-0.82] | **0.78**  [0.77-0.78] | **0.73**  [0.72-0.73] | **0.76**  [0.76-0.76] | **0.50**  [0.50-0.50] | **0.17**  [0.17-0.17] |

| ***30-day*** | **AUC** | **AP** | **PPV** | **NPV** | **F-1** | **MCC** | **Brier** |
| --- | --- | --- | --- | --- | --- | --- | --- |
| **LR** | 0.77  [0.77-0.77] | 0.80  [0.80-0.80] | **0.78**  [0.78-0.78] | 0.62  [0.61-0.62] | 0.69  [0.69-0.69] | 0.39  [0.39-0.40] | 0.20  [0.20-0.21] |
| **FF** | 0.77  [0.77-0.78] | **0.81**  [0.80-0.81] | 0.76  [0.76-0.76] | **0.65**  [0.65-0.66] | **0.73**  [0.72-0.73] | **0.41**  [0.41-0.42] | 0.20  [0.19-0.20] |
| **Xgb** | 0.77  [0.77-0.77] | **0.81**  [0.81-0.81] | **0.78**  [0.77-0.78] | 0.62  [0.62-0.63] | 0.70  [0.70-0.70] | 0.40  [0.40-0.41] | 0.20  [0.20-0.20] |

| ***Low risk*** | **AUC** | **AP** | **PPV** | **NPV** | **F-1** | **MCC** | **Brier** |
| --- | --- | --- | --- | --- | --- | --- | --- |
| **LR** | 0.84  [0.84-0.84] | 0.41  [0.41-0.41] | 0.39  [0.39-0.40] | 0.92  [0.92-0.92] | 0.45  [0.44-0.45] | 0.36  [0.35-0.36] | 0.13  [0.13-0.13] |
| **FF** | **0.85**  [0.85-0.85] | 0.44  [0.43-0.44] | **0.47**  [0.46-0.48] | 0.91  [0.91-0.91] | 0.40  [0.39-0.42] | 0.34  [0.33-0.35] | **0.10**  [0.10-0.10] |
| **Xgb** | **0.85**  [0.85-0.85] | **0.47**  [0.46-0.47] | 0.40  [0.39-0.41] | **0.93**  [0.93-0.93] | **0.46**  [0.45-0.46] | **0.37**  [0.36-0.38] | 0.11  [0.11-0.11] |

## Internal cross-validation of the overall European cohort

| ***ICU*** | **AUC** | **AP** | **PPV** | **NPV** | **F-1** | **MCC** | **Brier** |
| --- | --- | --- | --- | --- | --- | --- | --- |
| **LR** | 0.78  [0.77-0.79] | 0.73  [0.72-0.73] | **0.68**  [0.67-0.69] | 0.74  [0.73-0.75] | 0.68  [0.67-0.69] | 0.42  [0.41-0.43] | 0.19  [0.19-0.19] |
| **FF** | 0.78  [0.77-0.79] | 0.72  [0.71-0.73] | 0.65  [0.64-0.66] | **0.77**  [0.76-0.77] | **0.69**  [0.69-0.70] | 0.42  [0.40-0.43] | 0.20  [0.19-0.20] |
| **Xgb** | **0.81**  [0.80-0.81] | **0.75**  [0.75-0.76] | **0.68**  [0.67-0.69] | 0.75  [0.75-0.76] | **0.69**  [0.69-0.69] | **0.43**  [0.42-0.44] | **0.18**  [0.18-0.18] |

| ***30-day*** | **AUC** | **AP** | **PPV** | **NPV** | **F-1** | **MCC** | **Brier** |
| --- | --- | --- | --- | --- | --- | --- | --- |
| **LR** | 0.76  [0.76-0.77] | 0.71  [0.71-0.72] | **0.68**  [0.67-0.69] | 0.71  [0.70-0.71] | 0.65  [0.64-0.65] | 0.38  [0.37-0.39] | 0.20  [0.20-0.20] |
| **FF** | 0.76  [0.76-0.77] | 0.71  [0.70-0.72] | 0.66  [0.65-0.67] | **0.73**  [0.72-0.73] | 0.66  [0.65-0.67] | 0.38  [0.37-0.39] | 0.20  [0.20-0.20] |
| **Xgb** | **0.78**  [0.77-0.78] | **0.74**  [0.73-0.74] | **0.68**  [0.67-0.69] | **0.73**  [0.73-0.74] | **0.68**  [0.67-0.68] | **0.41**  [0.40-0.42] | **0.19**  [0.19-0.19] |

| ***Low risk*** | **AUC** | **AP** | **PPV** | **NPV** | **F-1** | **MCC** | **Brier** |
| --- | --- | --- | --- | --- | --- | --- | --- |
| **LR** | 0.86  [0.85-0.87] | 0.50  [0.48-0.53] | 0.49  [0.46-0.51] | **0.92**  [0.92-0.93] | 0.48  [0.46-0.50] | 0.40  [0.38-0.43] | 0.10  [0.09-0.10] |
| **FF** | 0.85  [0.84-0.87] | 0.51  [0.48-0.53] | 0.51  [0.47-0.54] | 0.91  [0.91-0.92] | 0.45  [0.43-0.47] | 0.38  [0.35-0.41] | 0.10  [0.09-0.10] |
| **Xgb** | **0.87**  [0.86-0.88] | **0.52**  [0.49-0.54] | **0.52**  [0.48-0.55] | **0.92**  [0.92-0.93] | **0.49**  [0.47-0.51] | **0.42**  [0.40-0.45] | 0.10  [0.09-0.10] |

## External validation of the European cohort on the patients from non-European ICUs

| ***ICU*** | **AUC** | **AP** | **PPV** | **NPV** | **F-1** | **MCC** | **Brier** |
| --- | --- | --- | --- | --- | --- | --- | --- |
| **LR** | 0.84  [0.84-0.84] | 0.84  [0.84-0.84] | 0.82  [0.82-0.82] | 0.65  [0.65-0.65] | 0.70  [0.69-0.70] | 0.46  [0.45-0.46] | 0.18  [0.18-0.18] |
| **FF** | 0.84  [0.84-0.85] | 0.84  [0.84-0.85] | 0.81  [0.81-0.82] | **0.71**  [0.70-0.72] | 0.76  [0.76-0.77] | 0.52  [0.52-0.53] | 0.17  [0.17-0.17] |
| **Xgb** | **0.89**  [0.89-0.89] | **0.88**  [0.88-0.88] | **0.84**  [0.84-0.85] | **0.71**  [0.71-0.72] | **0.77**  [0.76-0.77] | **0.55**  [0.55-0.56] | **0.15**  [0.15-0.15] |

| ***30-day*** | **AUC** | **AP** | **PPV** | **NPV** | **F-1** | **MCC** | **Brier** |
| --- | --- | --- | --- | --- | --- | --- | --- |
| **LR** | 0.83  [0.83-0.83] | 0.85  [0.85-0.85] | **0.82**  [0.82-0.82] | 0.68  [0.67-0.68] | 0.74  [0.73-0.74] | 0.50  [0.49-0.50] | 0.17  [0.17-0.18] |
| **FF** | 0.83  [0.83-0.83] | 0.84  [0.84-0.84] | 0.81  [0.80-0.81] | 0.72  [0.71-0.72] | 0.77  [0.77-0.77] | **0.53**  [0.52-0.53] | 0.17  [0.17-0.17] |
| **Xgb** | **0.86**  [0.86-0.86] | **0.87**  [0.86-0.87] | 0.81  [0.80-0.81] | **0.74**  [0.74-0.74] | **0.79**  [0.78-0.79] | 0.55  [0.54-0.55] | **0.16**  [0.15-0.16] |

| ***Low risk*** | **AUC** | **AP** | **PPV** | **NPV** | **F-1** | **MCC** | **Brier** |
| --- | --- | --- | --- | --- | --- | --- | --- |
| **LR** | 0.85  [0.85-0.85] | 0.59  [0.59-0.60] | 0.64  [0.63-0.64] | **0.80**  [0.80-0.80] | **0.42**  [0.41-0.43] | **0.33**  [0.33-0.34] | 0.15  [0.15-0.15] |
| **FF** | 0.85  [0.85-0.85] | 0.58  [0.58-0.58] | 0.64  [0.63-0.64] | 0.79  [0.79-0.80] | 0.39  [0.38-0.40] | 0.31  [0.31-0.32] | **0.14**  [0.14-0.14] |
| **Xgb** | **0.86**  [0.86-0.86] | **0.63**  [0.62-0.63] | **0.68**  [0.67-0.69] | 0.79  [0.79-0.79] | 0.37  [0.36-0.39] | 0.32  [0.31-0.33] | **0.14**  [0.14-0.14] |
