# Supplementary material for: COVID-19 machine learning model predicts outcomes in older patients from various European countries, between pandemic waves, and in a cohort of Asian, African, and American patients
Source: PLOS Digit Health. 2022 Nov 8;1(11):e0000136. doi: 10.1371/journal.pdig.0000136 (PMC9931233; doi:10.1371/journal.pdig.0000136)
Supplement: S5 Text — (DOCX) [file pdig.0000136.s005.docx]

# S5 Text – Variable ranking for the European and non-European cohort for each of the three outcomes of interest


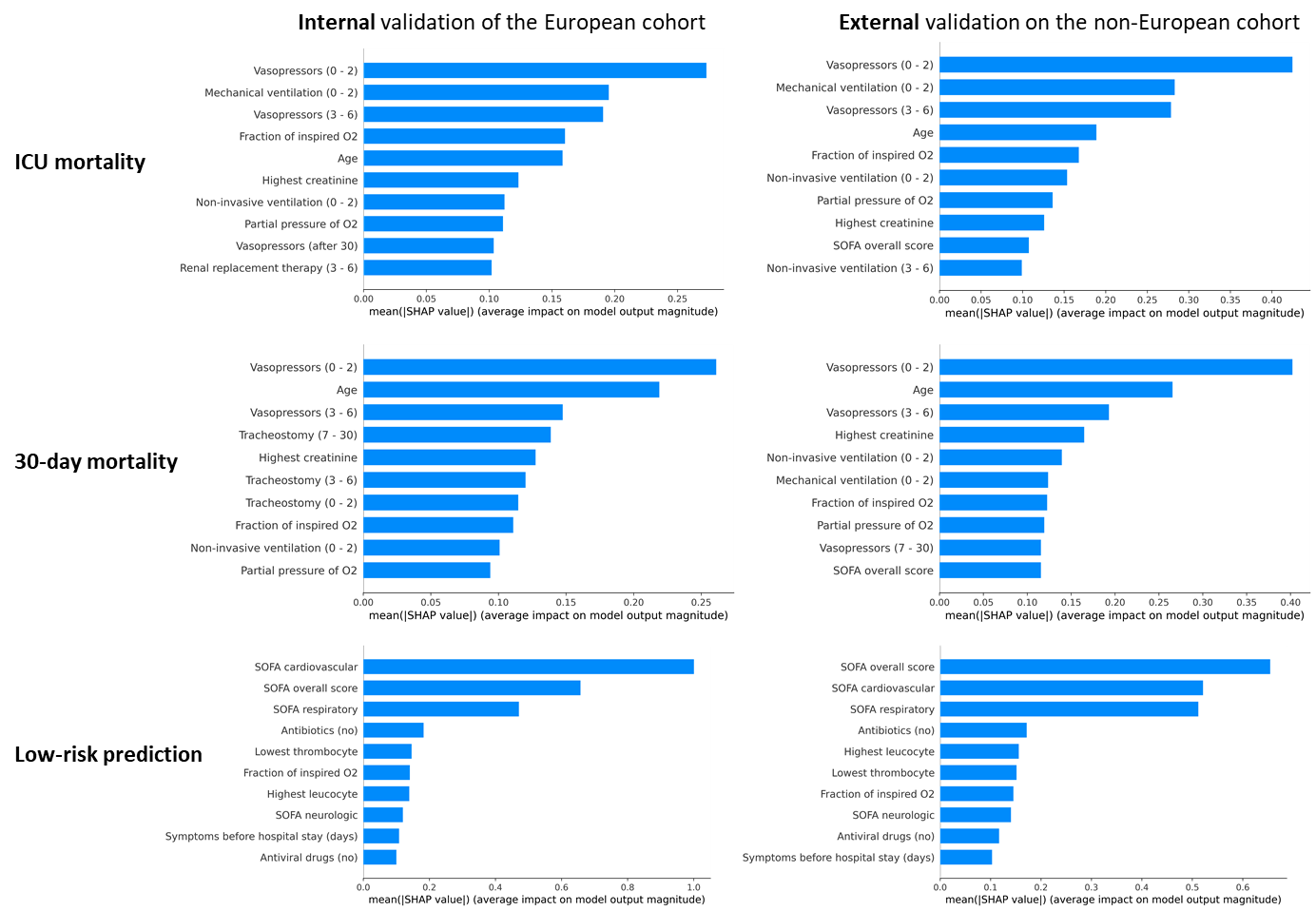


Fig A Variable ranking based on average SHAP values for each of the three outcomes for the model derived in the overall European cohort and validated in the external, non-European cohort.
