# Supplementary material for: COVID-19 machine learning model predicts outcomes in older patients from various European countries, between pandemic waves, and in a cohort of Asian, African, and American patients
Source: PLOS Digit Health. 2022 Nov 8;1(11):e0000136. doi: 10.1371/journal.pdig.0000136 (PMC9931233; doi:10.1371/journal.pdig.0000136)
Supplement: S6 Text — (DOCX) [file pdig.0000136.s006.docx]

S6 Text – Evaluation of generalisability of the model derived on the European cohort using each of the top-9 European countries (based on the number of ICU admissions) as the test cohort.

*Table A Model performance in predicting 30-day mortality in the European cohort of patients, evaluated on a single European country (shown in the first column based on ISO 3166-1, while England is represented as EN) and derived on the remaining European countries. Country and territory abbreviations are detailed in Appendix 9.*

| ***30-day*** | **AUC** | **AP** | **PPV** | **NPV** | **F-1** | **MCC** | **Brier** |
| --- | --- | --- | --- | --- | --- | --- | --- |
| **FR** | 0.79 | 0.68 | 0.63 | 0.78 | 0.66 | 0.42 | 0.19 |
| **ES** | 0.71 | 0.68 | 0.66 | 0.65 | 0.55 | 0.29 | 0.22 |
| **NL** | 0.74 | 0.64 | 0.60 | 0.73 | 0.58 | 0.32 | 0.20 |
| **DE** | 0.75 | 0.66 | 0.59 | 0.79 | 0.65 | 0.38 | 0.21 |
| **CH** | 0.79 | 0.77 | 0.70 | 0.73 | 0.70 | 0.42 | 0.19 |
| **DK** | 0.74 | 0.69 | 0.64 | 0.69 | 0.56 | 0.31 | 0.20 |
| **BE** | 0.83 | 0.78 | 0.79 | **0.79** | 0.67 | **0.54** | **0.16** |
| **EN** | **0.84** | **0.91** | **0.94** | 0.55 | **0.75** | 0.52 | 0.20 |
| **GR** | 0.80 | 0.83 | 0.75 | 0.67 | 0.73 | 0.41 | 0.18 |

*Table B Model performance in predicting ICU mortality in the European cohort of patients, evaluated on a single European country (shown in the first column based on ISO 3166-1) and derived on the remaining European countries. Country and territory abbreviations are detailed in Appendix 9.*

| **ICU** | **AUC** | **AP** | **PPV** | **NPV** | **F-1** | **MCC** | **Brier** |
| --- | --- | --- | --- | --- | --- | --- | --- |
| **FR** | 0.82 | 0.73 | 0.68 | 0.80 | 0.70 | 0.49 | 0.17 |
| **ES** | 0.71 | 0.69 | 0.70 | 0.62 | 0.61 | 0.32 | 0.22 |
| **NL** | 0.77 | 0.67 | 0.62 | 0.74 | 0.59 | 0.35 | 0.19 |
| **DE** | 0.83 | 0.80 | 0.66 | 0.80 | 0.72 | 0.47 | 0.17 |
| **CH** | 0.72 | 0.57 | 0.56 | 0.77 | 0.62 | 0.33 | 0.21 |
| **DK** | 0.82 | 0.74 | 0.67 | 0.82 | 0.69 | 0.49 | 0.18 |
| **BE** | **0.91** | 0.84 | 0.77 | **0.85** | 0.73 | **0.60** | **0.13** |
| **EN** | 0.86 | 0.89 | **0.87** | 0.67 | 0.77 | 0.54 | 0.17 |
| **GR** | 0.81 | **0.90** | 0.84 | 0.64 | **0.83** | 0.49 | 0.18 |

*Table C Model performance in predicting low-risk patients in the European cohort, evaluated on a single European country (shown in the first column based on ISO 3166-1) and derived on the remaining European countries. Country and territory abbreviations are detailed in Appendix 9.*

| **Low risk** | **AUC** | **AP** | **PPV** | **NPV** | **F-1** | **MCC** | **Brier** |
| --- | --- | --- | --- | --- | --- | --- | --- |
| **FR** | 0.86 | 0.53 | 0.56 | 0.86 | 0.42 | 0.34 | 0.11 |
| **ES** | 0.85 | 0.38 | 0.30 | 0.96 | 0.32 | 0.27 | **0.08** |
| **NL** | 0.83 | 0.34 | 0.30 | 0.95 | 0.40 | 0.33 | 0.12 |
| **DE** | 0.91 | 0.62 | 0.51 | 0.95 | 0.56 | **0.50** | 0.10 |
| **CH** | **0.92** | 0.56 | 0.28 | **0.98** | 0.40 | 0.38 | 0.09 |
| **DK** | 0.75 | 0.37 | 0.39 | 0.87 | 0.28 | 0.20 | 0.13 |
| **BE** | 0.80 | 0.67 | **0.74** | 0.66 | 0.35 | 0.27 | 0.18 |
| **EN** | 0.90 | **0.68** | 0.37 | 0.97 | 0.49 | 0.45 | 0.13 |
| **PT*** | 0.82 | 0.57 | 0.52 | 0.90 | **0.58** | 0.46 | 0.16 |

*Since there were no patients in the Greek (GR) cohort that met the criteria for low-risk patients, we considered the next available country, which was Portugal (PT).
