# Supplementary material for: COVID-19 machine learning model predicts outcomes in older patients from various European countries, between pandemic waves, and in a cohort of Asian, African, and American patients
Source: PLOS Digit Health. 2022 Nov 8;1(11):e0000136. doi: 10.1371/journal.pdig.0000136 (PMC9931233; doi:10.1371/journal.pdig.0000136)
Supplement: S7 Text — (DOCX) [file pdig.0000136.s007.docx]

S7 Text – Patients’ characteristics, including differences between the full set of variables, for the European and non-European cohort with respect to the three outcomes of interest (ICU and 30-day mortality and prediction of low-risk patients)

*Table A Patients’ characteristics for the European and non-European cohort with respect to 30-day mortality*

| **30-day mortality** | **European** | | | **Non-European** | | |
| --- | --- | --- | --- | --- | --- | --- |
| **Variable** | **Alive** | **Dead** | **p-value** | **Alive** | **Dead** | **p-value** |
| **Patients** | 1568 | 1290 | - | 281 | 335 | - |
| **Age**  **(year)** | 74  [72,77] | 76  [73,79] | <0.001 | 75  [72,80] | 77  [73,83] | <0.001 |
| **Sex (Female)** | 452  (28.8) | 375  (29.1) | 0.919 | 114  (40.6) | 130  (38.8) | 0.717 |
| **Weight**  **(kg)** | 81  [72,91] | 80  [72,90] | 0.041 | 78  [69.8,90] | 78  [70,87] | 0.708 |
| **Height**  **(cm)** | 171 [165,178] | 170 [164,176] | 0.017 | 165 [159,170] | 166 [160,173] | 0.142 |
| **BMI** | 27.7 [24.8,30.9] | 27.5 [24.7,30.9] | 0.427 | 27.9 [25.0,33.2] | 28.3 [25.6,31.1] | 0.722 |
| **SOFA overall score** | 4  [3,7] | 6  [4,9] | <0.001 | 4  [2,5] | 7  [5,10] | <0.001 |
| **Presence of diabetes** | 481  (30.8) | 471  (36.7) | 0.001 | 149  (53.2) | 194  (58.4) | 0.225 |
| **Ischemic heart disease** | 299  (19.3) | 327  (25.6) | <0.001 | 77  (27.6) | 93  (28.6) | 0.852 |
| **Renal comorbidity** | 175  (11.2) | 273  (21.3) | <0.001 | 29  (10.4) | 80  (24.3) | <0.001 |
| **Arterial hypertension** | 1025  (65.6) | 861  (66.9) | 0.483 | 172  (61.6) | 235  (71.0) | 0.019 |
| **Pulmonary disease** | 337  (21.5) | 305  (23.8) | 0.164 | 45  (16.1) | 61  (18.8) | 0.457 |
| **Congestive heart failure** | 178  (11.5) | 230  (18.1) | <0.001 | 32  (11.6) | 49  (14.9) | 0.286 |
| **Mechanical ventilation** | 1057  (67.4) | 1064  (82.5) | <0.001 | 69  (24.6) | 271  (80.9) | <0.001 |
| **Vasopressors** | 997  (63.6) | 1085  (84.1) | <0.001 | 37  (13.2) | 185  (55.2) | <0.001 |
| **Renal replacement therapy** | 180  (11.5) | 282  (21.9) | <0.001 | 17  (6.0) | 51  (15.2) | <0.001 |
| **Non-invasive ventilation** | 401  (25.6) | 344  (26.7) | 0.536 | 73  (26.0) | 157  (46.9) | <0.001 |
| **Tracheostomy** | 378  (24.1) | 161  (12.5) | <0.001 | 17  (6.0) | 10  (3.0) | 0.098 |
| **ICU LoS**  **(day)** | 15  [7,32] | 11  [6,17] | <0.001 | 6  [4,10] | 7  [4,10] | 0.767 |

*Table B Patients’ characteristics for the European and non-European cohort with respect to identifying low-risk patients*

| **Low-risk** | **European** | | | | **non-European** | | | |
| --- | --- | --- | --- | --- | --- | --- | --- | --- |
| **Variable** | **Low risk** | **High risk (survivors)** | **High risk (non-survivors)** | **p-value** | **Low risk** | **Alive** | **Dead** | **p-value** |
| **Patients** | 371 | 1203 | 1284 | - | 157 | 127 | 332 | - |
| **Age**  **(year)** | 75 [72.5,79] | 74 [72,77] | 75 [72,79] | <0.001 | 76 [73,80] | 75 [72,80] | 77 [73,83] | 0.006 |
| **Sex (Female)** | 116 (31.3) | 361 (30.0) | 350 (27.3) | 0.182 | 65  (41.4) | 50  (39.4) | 129 (38.9) | 0.864 |
| **Weight**  **(kg)** | 80 [70,90] | 81 [72,91] | 80 [72,90] | 0.016 | 80 [67,95.5] | 77 [70,85] | 78 [70,87] | 0.424 |
| **Height**  **(cm)** | 170 [164,176] | 170 [165,178] | 170 [165,177] | 0.087 | 160 [154,166] | 167 [160,172] | 166 [160,173] | <0.001 |
| **BMI** | 27.1 [24.2,30.5] | 27.7 [25,31] | 27.5 [24.7,30.8] | 0.1 | 31.2 [25.6,38] | 27.4 [24.8,30.5] | 28.3 [25.6,31.1] | 0.003 |
| **SOFA overall score** | 3  [2,4] | 5  [3,8] | 6  [4,9] | <0.001 | 3  [2,4] | 5  [3,8] | 7  [5,10] | <0.001 |
| **Presence of diabetes** | 113 (30.6) | 377 (31.4) | 462 (36.2) | 0.02 | 87  (55.4) | 69  (54.8) | 187 (56.8) | 0.908 |
| **Ischemic heart disease** | 90  (24.3) | 228 (19.2) | 308 (24.3) | 0.005 | 39  (24.8) | 39  (31.2) | 92  (28.6) | 0.484 |
| **Renal comorbidity** | 51  (13.8) | 142 (11.8) | 255 (20.0) | <0.001 | 5  (3.2) | 24  (19.2) | 80  (24.5) | <0.001 |
| **Arterial hypertension** | 248 (67.2) | 781 (65.1) | 857 (66.9) | 0.572 | 87  (55.4) | 87  (69.0) | 233 (71.3) | 0.002 |
| **Pulmonary disease** | 83  (22.4) | 258 (21.5) | 301 (23.6) | 0.442 | 21  (13.4) | 26  (20.6) | 59  (18.4) | 0.238 |
| **Congestive heart failure** | 54  (14.7) | 149 (12.5) | 205 (16.2) | 0.032 | 14  (9.0) | 19  (15.3) | 48  (14.8) | 0.169 |
| **ICU LoS**  **(day)** | 5  [2,7.5] | 16  [8,31] | 14  [7,22] | <0.001 | 5  [3,9] | 8  [4.5,15] | 7  [4,11] | <0.001 |
