# Supplementary figures and images for: COVID-19 machine learning model predicts outcomes in older patients from various European countries, between pandemic waves, and in a cohort of Asian, African, and American patients
Source: PLOS Digit Health. 2022 Nov 8;1(11):e0000136. doi: 10.1371/journal.pdig.0000136 (PMC9931233; doi:10.1371/journal.pdig.0000136)

# S8 Text – Cohort selection diagram


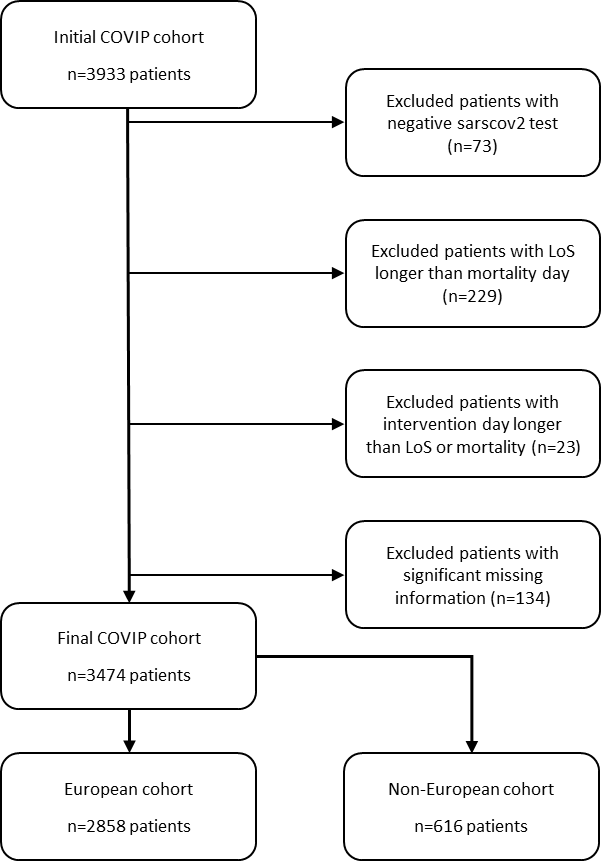

Supplement: S8 Text — (DOCX) [file pdig.0000136.s008.docx]
