# Supplementary material for: COVID-19 machine learning model predicts outcomes in older patients from various European countries, between pandemic waves, and in a cohort of Asian, African, and American patients
Source: PLOS Digit Health. 2022 Nov 8;1(11):e0000136. doi: 10.1371/journal.pdig.0000136 (PMC9931233; doi:10.1371/journal.pdig.0000136)
Supplement: S9 Text — (DOCX) [file pdig.0000136.s009.docx]

S9 Text – Abbreviations of the countries and territories

| **AT** | Austria | **LY** | Libya |
| --- | --- | --- | --- |
| **BE** | Belgium | **MA** | Morocco |
| **CH** | Switzerland | **MX** | Mexico |
| **CO** | Colombia | **NL** | Netherlands |
| **DE** | Germany | **NO** | Norway |
| **DK** | Denmark | **OM** | Oman |
| **EG** | Egypt | **PS** | Palestine |
| **EN** | England (UK) | **PK** | Pakistan |
| **ES** | Spain | **PO** | Poland |
| **FR** | France | **PS** | Palestine |
| **GR** | Greece | **PT** | Portugal |
| **IE** | Ireland | **RO** | Romania |
| **IL** | Israel | **SA** | Saudi Arabia |
| **IN** | India | **SD** | Sudan |
| **IQ** | Iraq | **SY** | Syrian Arab Republic |
| **IR** | Iran | **TR** | Turkey |
| **IT** | Italy | **US** | United States of America |
| **JO** | Jordan | **WL** | Wales (UK) |
| **LB** | Lebanon | **YE** | Yemen |
